# ReconUNet: Learning to Reconstruct a Clean Covariance Matrix Under Low SNR, Multipath, and Array Imperfections

**DOI:** 10.3390/s26217041

**Published:** 2026-11-07

**Authors:** Itamar Almog, Anthony J. Weiss

**Affiliations:** School of Electrical Engineering, Tel Aviv University, Tel Aviv 6997801, Israel; tony@tauex.tau.ac.il

**Keywords:** direction-of-arrival, DoA estimation, array signal processing, deep learning, covariance reconstruction, subspace methods, MUSIC, ESPRIT, array imperfections, coherent multipath

## Abstract

Direction-of-arrival (DoA) estimation degrades under low signal-to-noise ratio, coherent multipath, and array imperfections. Coherent replicas are especially damaging because they collapse the rank of the signal covariance. We propose ReconUNet, a U-Net that maps a stack of multi-lag autocorrelations to a covariance matrix. It is trained with a single reconstruction loss toward the closed-form clean covariance on the nominal manifold, and its output feeds unmodified classical estimators. In simulations with an eight-element array, we compare it to six classical estimators, spatial smoothing, and three learned frontends trained on the same corpus. With a Root-MUSIC backend, ReconUNet attains a pooled RMSE of 4.23° on the held-out split, against 7.91° for the best learned baseline and 12.43° for Root-MUSIC on the sample covariance. It has the lowest error for every source count, and one trained model serves MUSIC, Root-MUSIC, ESPRIT, and Unitary-ESPRIT. The network acts as a learned regularizer of the covariance, not as an array calibrator. Its multi-lag input depends on the temporal correlation of the sources. When trained at one bandwidth, it fails for wideband sources; when trained over a range of bandwidths, it is the most accurate method tested across that range. Code and weights are released with the paper.

## 1. Introduction

### 1.1. Background and Classical Methods

Direction-of-arrival (DoA) estimation is a cornerstone of array signal processing, with applications in wireless communications, radar, sonar, acoustics, and remote sensing [1,2]. An array acts as a spatial filter, separating signals that overlap in frequency but arrive from different directions [3]. This capability underpins smart antennas, massive MIMO, passive sensing, and localization, where accurate DoA estimates translate into link reliability, interference suppression, and tracking.

Classical estimators fall into two families. *Beamformers* scan for power across angles. The conventional (Bartlett) beamformer performs steering and averaging, and its resolution is limited by the array beampattern [4]; Capon’s minimum variance distortionless response (MVDR) beamformer sharpens the response by minimizing the output power away from the candidate direction [5]. *Subspace methods* recast DoA estimation as geometry on the array manifold. MUSIC estimates the noise subspace from the sample covariance and searches for steering vectors orthogonal to it [6]. Root-MUSIC replaces the search with polynomial rooting for uniform linear arrays (ULAs) [7]. ESPRIT avoids the search altogether by exploiting the rotational invariance between two subarrays [8], and Unitary-ESPRIT adds forward–backward averaging in a real-valued formulation, which improves accuracy for correlated sources [9,10]. The Cramér–Rao lower bound (CRLB) [3,11] and the Ziv–Zakai bound [12] are the references against which these estimators are judged.

Subspace methods rest on a structural assumption, namely, that the array covariance splits into a *K*-dimensional signal subspace and an (N−K)-dimensional noise subspace. Four common conditions violate this assumption: limited snapshots make the sample covariance noisy; low SNR shrinks the gap between the signal and noise eigenvalues; coherent multipath collapses the rank of the signal covariance so that fewer than *K* dominant eigenvalues remain; and array imperfections (gain/phase errors, sensor position errors, and mutual coupling) distort the manifold so that the steering vectors assumed by the estimator no longer match the data. Remedies exist for each condition in isolation; for instance, spatial smoothing [13,14,15] restores the rank at the cost of aperture, while self-calibration [16] estimates the coupling matrix. However, these remedies are scenario-specific, and each consumes degrees of freedom that cannot be spared for a small array.

### 1.2. Learning-Based DoA Estimation

Over the past decade, deep learning has become a general tool for learning representations directly from data [17,18]. Model-based deep learning has shown how to combine learned components with the inductive biases of classical signal processing [19]. Surveys of learned beamforming have reported substantial gains in detection, localization, and interference suppression when learning is aligned with the physics [20]. Learning-based DoA estimators fall into three groups.

*Spectrum learners* output a spatial spectrum, which is then peak-searched. Early work learned spectra from engineered features, and showed robustness to gain/phase drift, position errors, and mutual coupling when trained with designed imperfections [21]. At low SNR, convolutional networks have been shown to sharpen peaks and suppress spurious responses relative to classical spectra [22]. DeepMUSIC learns MUSIC-like spectra by classification over angular subregions [23], and SDOA-Net is trained at the snapshot level under joint imperfections while keeping spectrum formation outside the network [24]. These models are fast and simple to postprocess, but their accuracy degrades under train–test manifold mismatch, and they inherit on-grid and peak association errors.

*Statistics learners* keep the physics and learn the second-order statistics required by classical methods. SubspaceNet takes multi-lag autocorrelations as input, learns a covariance surrogate, and is trained end-to-end through a differentiable Root-MUSIC on angle labels, so that the signal and noise subspaces remain separable under coherence and array mismatch [25]. DA-MUSIC learns MUSIC-internal quantities (a pseudo-covariance and the peak structure of the spectrum) in a deep-augmented architecture [26], and DCD-MUSIC extends this approach to near-field localization of multiple sources with a cascaded differentiable MUSIC [27]. Covariance reconstruction hybrids first denoise the covariance, then attach a direct angle head [28]. These methods retain the structure of classical estimators, but at the cost of embedding a particular estimator in the training loop.

*Direct predictors* regress or classify the angles themselves. Convolutional networks operating on covariance “images” perform well at low SNR, often for a fixed small number of sources and with discretization effects when the task is cast as classification [29]. Transformer variants tokenize the spatial covariance and use transfer learning to adapt from ideal to imperfect arrays [30]. Recent work has extended direct prediction to three-dimensional estimation with deep convolutional networks [31], and through spatial attention to angle estimation across diverse array configurations [32]. Direct predictors offer end-to-end simplicity; how they handle a variable number of sources, source coherence, and unseen array mismatch remains an open question.

### 1.3. This Work: Positioning and Contributions

This paper proposes an estimator-agnostic frontend that reconstructs a clean covariance from noisy and imperfect measurements, then passes it to an unmodified classical estimator. Following the “learn the statistics, keep the physics” principle, the network takes a stack of multi-lag autocorrelations as input, while a U-Net backbone [33] with a Hermitian covariance head predicts the covariance. Two design choices distinguish ReconUNet from the statistics learners above (Table 1). First, the supervision target is the closed-form clean covariance on the nominal array manifold, which exists for any scene once the geometry is specified. No angle labels are propagated through an estimator, no estimator is inside the training loop, and the loss is a single reconstruction term. Second, the output is a covariance matrix, so the same trained weights serve spectrum scanning estimators (Bartlett, MVDR, MUSIC), rooting estimators (Root-MUSIC), and rotational invariance estimators (ESPRIT, Unitary-ESPRIT) without retraining.

The first submitted version of this work also predicted an explicit eigendecomposition, with dedicated eigenvalue and eigenvector heads trained by a four-term loss. A component-wise ablation showed that these heads and the auxiliary loss terms do not help; the covariance reconstruction objective and the multi-lag input carry the method. The network proposed here is a simpler covariance-only one. The variant with eigendecomposition heads, denoted ReconUNet-EVD, is described in Appendix A and kept in the results as a reference in order to show the cost of its additional components.

The proposed network does not estimate the imperfections of a particular array, and is not a calibration method; it learns the expected clean covariance given the corrupted input, which is averaged over the distribution of imperfections and source statistics seen in training (Section 3.5). In the simulated scenarios we study here, its benefit is robustness to coherent multipath when the array imperfections are unknown, meaning that classical estimators fed with its output no longer fail catastrophically. Its limits follow from the same mechanism. The result depends on how well the training distribution covers the test conditions, which we examine for the imperfection level, SNR, number of snapshots, source separation, and source bandwidth. All experiments are simulations with parametric imperfection models; transfer to measured arrays is not demonstrated in this paper.

The contributions of this work are as follows:

- A covariance reconstruction frontend for classical DoA estimators, consisting of a U-Net that maps multi-lag autocorrelations to a Hermitian covariance and is trained with a single reconstruction loss toward closed-form targets on the nominal manifold, with no estimator in the training loop.
- An analysis of why the non-zero lags make coherent replicas separable when the sample covariance does not (Section 3.2), along with measurements of where this holds. The benefit requires a source coherence time of several samples; for wideband or white sources, it depends on whether the training distribution covers them (Section 5.7).
- A controlled simulation study, on identical data and with identical training budgets, against three learned front-ends (SubspaceNet, DA-MUSIC, and a covariance input transformer regressor), six classical estimators, and forward–backward spatial smoothing. The study covers source count, SNR, coherent multipath, two levels of array imperfection, snapshot count, source separation, and source bandwidth, and also includes a component-wise ablation.

The rest of this paper is organized as follows: Section 2 presents the array, multipath, and imperfection models along with the problem statement; Section 3 describes the proposed frontend; Section 4 details the data, baselines, protocol, and metrics; Section 5 reports the results, ablation, robustness analysis, and computational cost; finally, Section 6 concludes the paper.

## 2. Signal Model

**Figure 1:**
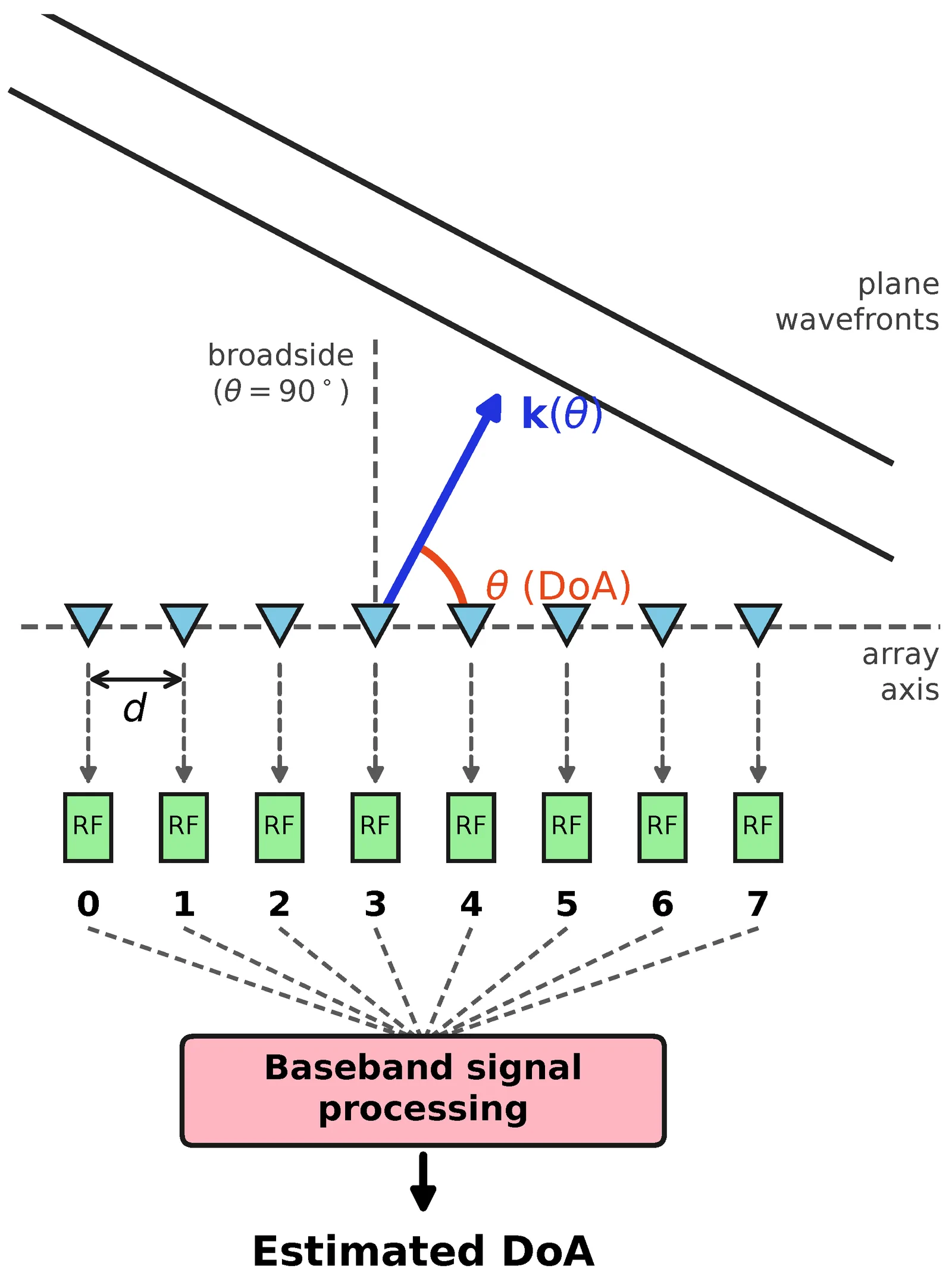
Uniform linear array (ULA) geometry and angle convention. Element spacing is *d*; the DoA θ is measured from the array axis (broadside $=90^{\circ }$). The figure is drawn for the N=8 array used in this paper (elements 0,…,7). Solid black lines: plane wavefronts; blue arrow: wave vector k(θ) of (1); red arc: DoA θ; dashed lines: array axis, broadside direction, and signal paths from each element through its RF chain (green) to baseband processing.

This section introduces the three models on which the paper is built: the ideal narrowband array model, the specular multipath model that produces coherent replicas, and the array imperfection model. For each model, we state the assumption and how it breaks a subspace estimator, as these failure modes are what the proposed frontend is trained to mitigate. Figure 1 shows the geometry and angle convention.

### 2.1. Ideal Narrowband Model

Consider an array of *N* sensors with nominal positions $r_{n}\in R^{2}$, n=0,…,N−1. At snapshot t∈{1,…,T}, the array output is $x\left(t\right)\in C^{N}$ and the data matrix is $X=\left[x\left(1\right),\ldots ,x\left(T\right)\right]\in C^{N\times T}$. There are *K* far-field emitters with DoAs $\theta =[\theta_{1},\ldots ,\theta_{K}]$, measured from the array axis. With wave vector

(1) $$k\left(\theta \right)=\frac{2\pi }{\lambda }\left[\begin{array}{c} \cos\theta \\ \sin\theta \end{array}\right],$$

the steering vector and steering matrix are

(2) $${\left[a(\theta )\right]}_{n}=e^{\;j\;k{\left(\theta \right)}^{T}r_{n}},\;A\left(\theta \right)=\left[a\left(\theta_{1}\right),\ldots ,a\left(\theta_{K}\right)\right]\in C^{N\times K}.$$

With source vector $s\left(t\right)\in C^{K}$ and spatially white noise $n\left(t\right)∼\mathrm{CN}\left(0,\sigma_{n}^{2}I_{N}\right)$ independent of the sources, the snapshot model is

(3) x(t)=A(θ)s(t)+n(t).

We use three covariance objects throughout, which are consistently referred to as follows: the *ensemble* covariance and its decomposition are

(4) $$R_{x}≜E\left\{x\left(t\right)x^{H}\left(t\right)\right\}=A\left(\theta \right)\;R_{s}\;A^{H}\left(\theta \right)+\sigma_{n}^{2}I_{N},$$

with source covariance $R_{s}≜E\left\{s\left(t\right)s^{H}\left(t\right)\right\}⪰0$; the *sample* covariance is ${\hat{R}}_{x}=\frac{1}{T}XX^{H}$; and the *reconstructed* covariance produced by the network is $\hat{R}$ (Section 3). When $\mathrm{rank}(R_{s})=K$, the ensemble covariance splits into a *K*-dimensional signal subspace spanned by the columns of A and an (N−K)-dimensional noise subspace. Every subspace estimator exploits this structure.

### 2.2. Specular Multipath and Coherent Sources

In addition to line of sight, a dominant emitter can reach the array through several mirror-like reflections. Each replica *k* has an excess path length $\Delta \ell_{k}$, a group delay $\tau_{k}=\Delta \ell_{k}/c$, a path loss $L_{k}$ in dB with linear gain $\alpha_{k}=10^{-L_{k}/20}$, and a reflection phase $\phi_{k}$ that depends on the material and polarization. At the carrier frequency $f_{c}$, the complex baseband gain of the replica is

(5) $$g_{k}=\alpha_{k}\;e^{j\phi_{k}}\;e^{-j2\pi f_{c}\tau_{k}}.$$

Whether a replica is coherent with the direct path depends on the source bandwidth. Let *B* be the essential baseband bandwidth of the direct signal s(t), and let $r\left(\Delta \right)≜E\{s\left(t\right)s^{*}\left(t-\Delta \right)\}$ be its autocorrelation. The normalized envelope γ(τ)≜|r(τ)|/r(0) decays on the scale of the coherence time $T_{\mathrm{coh}}\approx 1/B$, so replicas with $0\leq \tau_{k}≲1/B$ remain highly correlated with the direct path. This is the regime we model here. In our data, the sources are band-limited to 5% of the sampling rate (10% of the Nyquist band), which gives a coherence time of about 20 snapshots, and the replica delays are drawn within it (Section 4).

To see why coherence is destructive, consider one direct source at angle θ and one replica at angle $\theta^{\mathrm{mp}}$ with delay τ and gain *g*. Writing a≜a(θ) and $b≜a(\theta^{\mathrm{mp}})$, the snapshot is

(6) x(t)=as(t)+bgs(t−τ)+n(t),

and with $P≜E\{|s\left(t\right){|}^{2}\}$, the ensemble covariance expands to

(7) $$\begin{array}{cc} R_{x} & =P\;aa^{H}+{\left|g\right|}^{2}P\;bb^{H}\\ \; & \;+g\;r\left(\tau \right)\;ab^{H}+g^{*}r^{*}\left(\tau \right)\;ba^{H}+\sigma_{n}^{2}I_{N}. \end{array}$$

Well inside the coherence window, r(τ)=Pγ with $\gamma \approx e^{j\varphi }$, |γ|≈1, and the signal part of (7) factorizes as

(8) $$R_{x,\mathrm{sig}}=P\;\left(a+g\gamma \;b\right){\left(a+g\gamma \;b\right)}^{H}≜P\;v_{\mathrm{eff}}v_{\mathrm{eff}}^{H}.$$

The signal covariance has rank one. A delayed narrowband replica is a scaled and phase-rotated copy of the same waveform, so there is only one independent temporal degree of freedom, and the spatial covariance is built from a single effective steering vector $v_{\mathrm{eff}}$ that lies on neither a(θ) nor $a(\theta^{\mathrm{mp}})$. A subspace estimator told that K=1 then estimates the direction of $v_{\mathrm{eff}}$, which is biased. When told the true number of arrivals, it finds too few dominant eigenvalues, and its noise subspace is contaminated. Either way, the estimate can fail catastrophically rather than degrading gracefully; this failure mode dominates the tails of the error distributions in Section 5. Our data confirm the above analysis. On coherent scenes, the second signal eigenvalue of the sample covariance is 2.5% of the first on average.

### 2.3. Array Imperfections

The nominal ULA has positions $r_{n}^{\left(0\right)}={\left[nd,\;0\right]}^{T}$, so that with d=λ/2, the nominal steering entry is ${\left[a_{0}\left(\theta \right)\right]}_{n}=\exp\left(j\pi n\cos\theta \right)$. We model three departures from this, each drawn independently per scene.

*Position errors.* Each element is displaced by $\varepsilon_{n}∼N\left(0,\sigma_{\mathrm{pos}}^{2}I_{2}\right)$, giving the actual positions $r_{n}=r_{n}^{\left(0\right)}+\varepsilon_{n}$ and the position-perturbed manifold ${\left[a_{\mathrm{pos}}\left(\theta \right)\right]}_{n}=\exp\left(jk{\left(\theta \right)}^{T}r_{n}\right)$. The distortion is direction-dependent and produces steering mismatch, bias, and broadening of the main lobe.

*Gain and phase errors.* Hardware variability across channels is modeled by the diagonal matrix

(9) $$G=\mathrm{diag}{\left\{g_{n}e^{\;j\phi_{n}}\right\}}_{n=0}^{N-1},\;g_{n}∼U\left[1-\delta_{g},1+\delta_{g}\right],\;\phi_{n}∼U\left[-\delta_{\phi },\delta_{\phi }\right],$$

which introduces amplitude/phase imbalance and breaks the nominal symmetry of the array.

*Mutual coupling.* Electromagnetic coupling between elements is captured by a reciprocal distance-ordered Toeplitz matrix

(10) $$M=I_{N}+E,\;E\left[i,j\right]=\left\{\begin{array}{cc} 0, & i=j,\\ c_{\left|i-j\right|}, & i\neq j, \end{array}\right.$$

with geometrically decaying off-diagonals $c_{k}=\gamma_{c}^{\;k}v_{k}$, $\gamma_{c}=\rho \;e^{j\varphi_{c}}$, $v_{k}∼U\left[1-\delta /2,\;1+\delta /2\right]$, k=1,…,N−1. Coupling mixes the sensor outputs, so that even for uncorrelated sources the columns of the effective steering matrix are not orthogonal under the noise metric. This shrinks the signal–noise eigengap and flattens MUSIC-type spectra. Coupling also destroys the centro-Hermitian symmetry of the array, weakening the rotational invariance structure on which Unitary-ESPRIT and forward–backward averaging rely.

Collecting the hardware effects into the composite operator H=MG, the distorted steering vectors and matrix are $\tilde{a}\left(\theta \right)=H\;a_{\mathrm{pos}}\left(\theta \right)$ and $\tilde{A}\left(\theta \right)=\left[\tilde{a}\left(\theta_{1}\right),\ldots ,\tilde{a}\left(\theta_{K}\right)\right]$, and the observation and covariance become

(11) $$x\left(t\right)=\tilde{A}\left(\theta \right)\;s\left(t\right)+n\left(t\right),\;R_{x}=\tilde{A}\;R_{s}\;{\tilde{A}}^{H}+\sigma_{n}^{2}I_{N}.$$

The numerical values of $\left(\sigma_{\mathrm{pos}},\delta_{g},\delta_{\phi },\rho ,\varphi_{c},\delta \right)$ used in training and in the two evaluation presets are given in Section 4.

### 2.4. Problem Formulation

Given *T* narrowband far-field snapshots X that obey (11), possibly with coherent replicas as in Section 2.2, the task is to estimate the respective DoAs θ of the *K* direct sources. Throughout this paper, *K* counts direct sources only (coherent replicas are not counted); it is assumed that *K* is known and supplied identically to every estimator, whether learned or classical. Let G denote an estimator (beamforming scan, MUSIC, Root-MUSIC, ESPRIT, etc.) producing $\hat{\theta }=G\left({\hat{R}}_{x}\right)$. Accuracy is measured by the permutation-invariant set error

(12) $${\mathrm{RMSE}}^{2}\left(\hat{\theta },\theta \right)=\frac{1}{K}\min_{P\in \Pi_{K}}∥\hat{\theta }-P\theta {∥}_{2}^{2},$$

where $\Pi_{K}$ is the set of K×K permutation matrices; the error is reported in degrees. The regimes of interest are low SNR, coherent multipath with small delays, and array imperfections. These degrade the eigengap as well as the separability of spectral peaks, and are the conditions under which all estimators are assessed in Section 5.

## 3. Proposed Method

### 3.1. Overview

**Figure 2:**
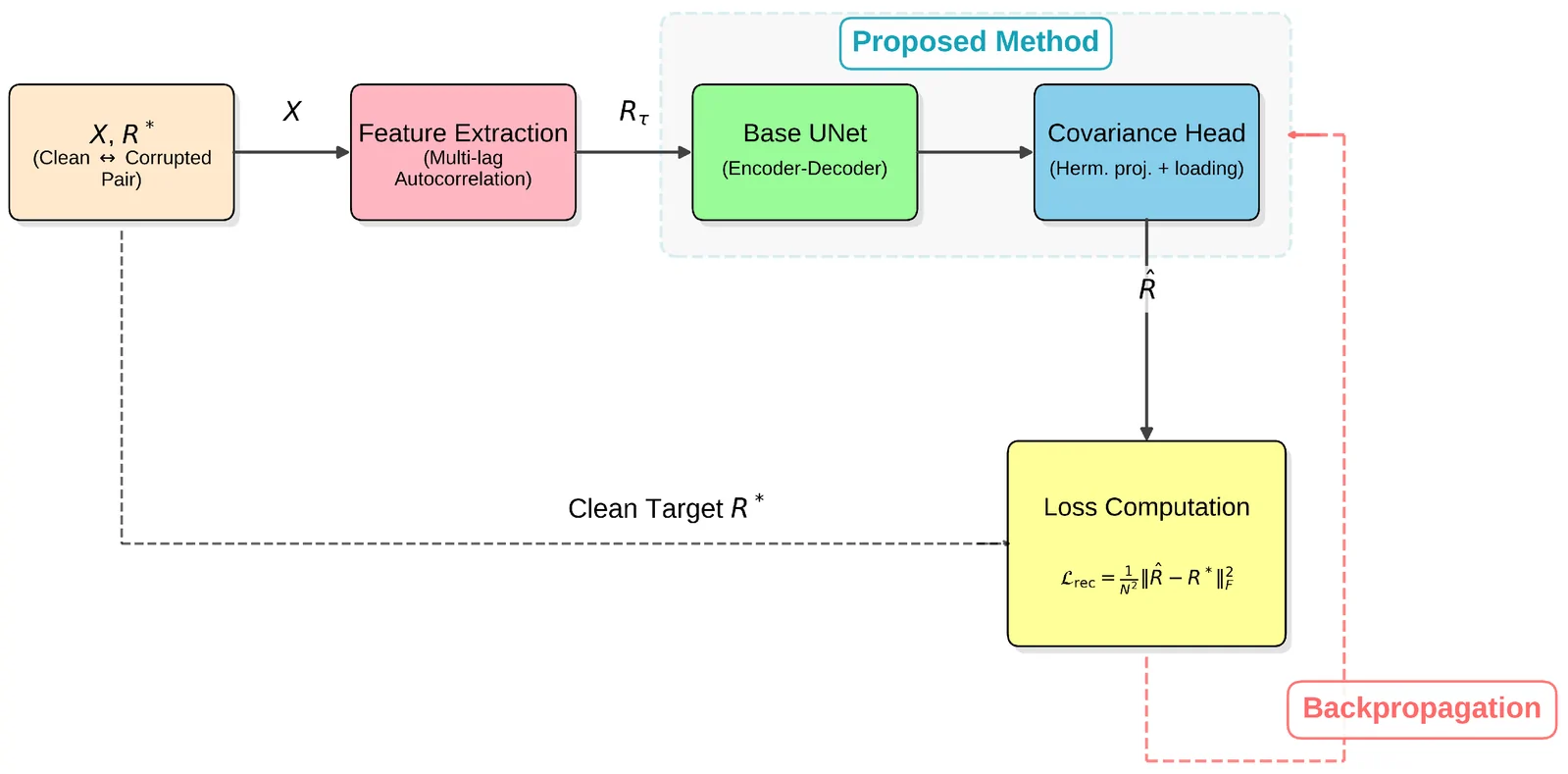
Training: Corrupted snapshots are converted to a multi-lag autocorrelation stack, the network predicts a covariance, and the loss compares it with the closed-form clean target. Solid arrows: forward data flow; black dashed arrow: clean target; red dashed arrow: backpropagation of the loss gradient to the network. In the figure, *X* is the snapshot matrix X, $R_{\tau }$ the multi-lag autocorrelation stack, $\hat{R}$ the reconstructed covariance $\hat{R}$, and $R^{*}$ the clean target $R^{★}$ of (17); the asterisk is part of the symbol.

**Figure 3:**
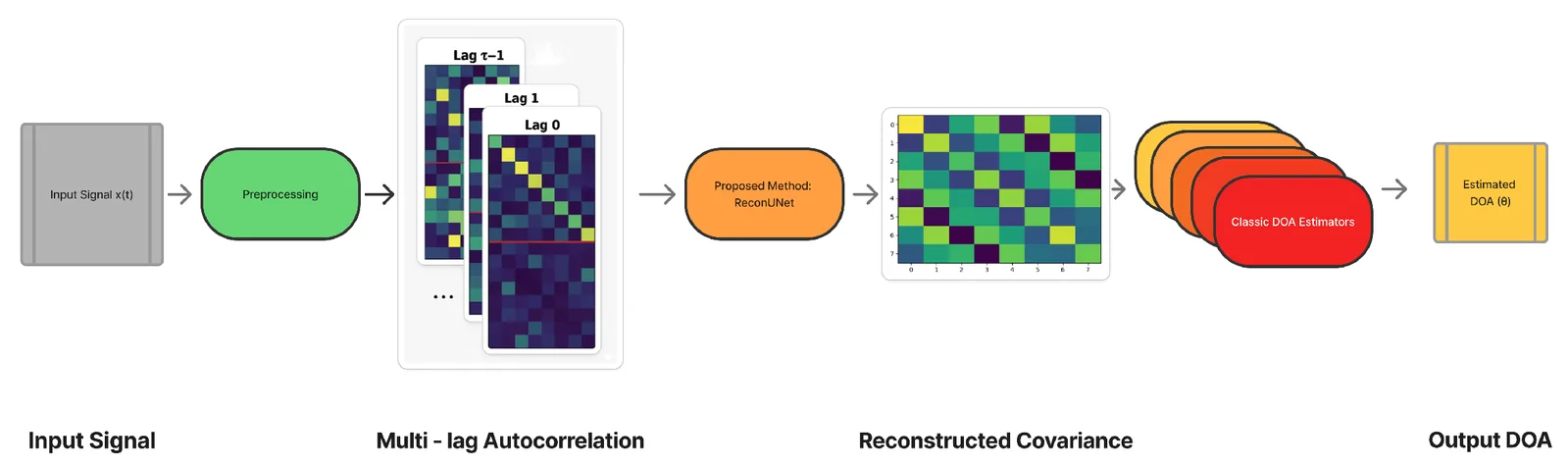
Inference: Multi-lag autocorrelation → ReconUNet → reconstructed covariance → unmodified classical DoA estimator. The stacked matrices show the lags ℓ=0,1,…,τ−1 of (13); the ellipsis stands for the intermediate lags ℓ=2,…,τ−2. Each lag is shown with its real part above its imaginary part (separated by the red line), as in (14); colors show illustrative matrix values. The stacked estimator boxes indicate that any classical estimator (MUSIC, Root-MUSIC, ESPRIT, Unitary-ESPRIT) can be applied to the same output; the overlaps are intentional.

**Figure 4:**
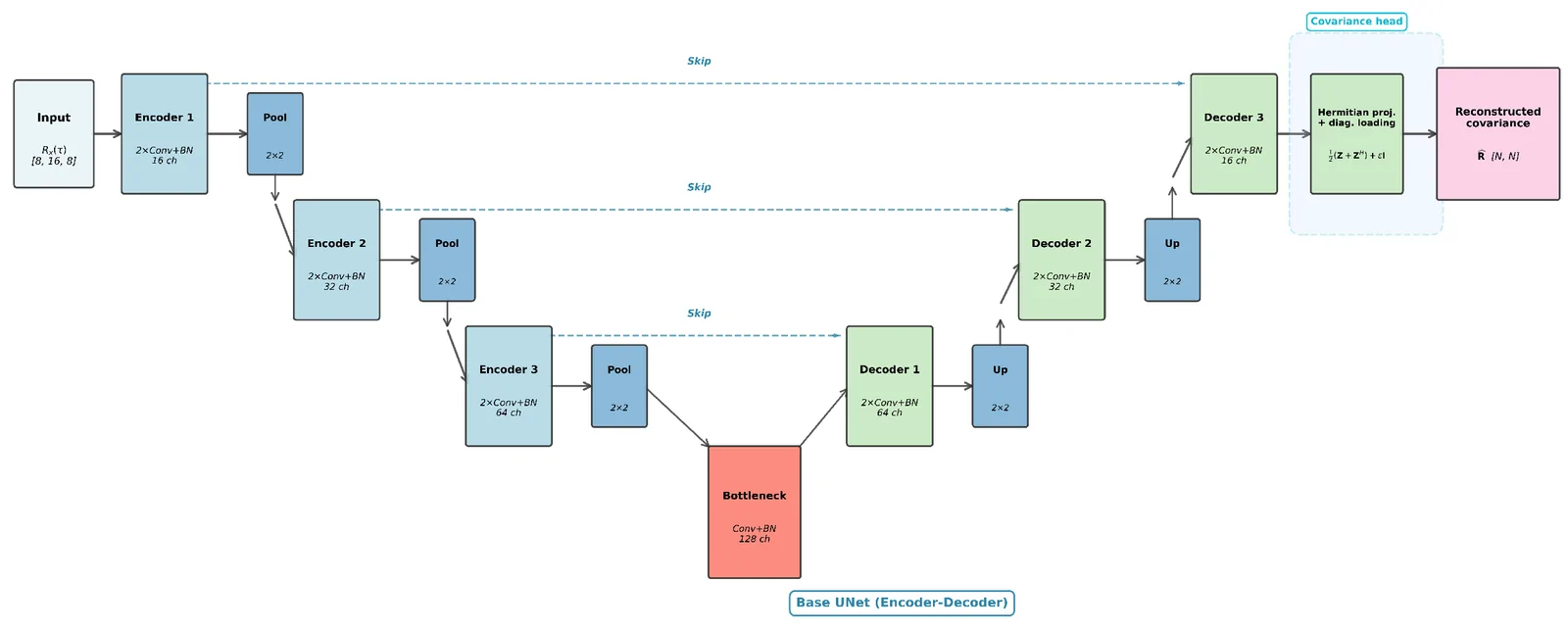
Architecture: Three-level U-Net with skip connections on the lag–sensor grid, followed by the covariance head (Hermitian projection and diagonal loading). The eigendecomposition heads of the ReconUNet-EVD variant (Appendix A) are not part of the proposed network. “[8, 16, 8]” is the shape of the input tensor (τ×2N×N with τ=8, N=8), not a citation, and “[N, N]” is the output size. Light blue: encoder blocks; dark blue: pooling and upsampling; red: bottleneck; green: decoder blocks and covariance head; pink: output. Solid arrows: data flow; dashed arrows: skip connections.

ReconUNet maps raw snapshots to a single output, consisting of a Hermitian positive-definite estimate $\hat{R}$ of the clean covariance. It does not replace classical estimators, only their input, causing MUSIC, Root-MUSIC, ESPRIT, and related estimators to operate closer to their ideal regime under low SNR, coherent multipath, and array imperfections. The name ReconUNet combines these two ideas: reconstruction of the clean covariance, with U-Net as the backbone. Figure 2 shows the training flow, Figure 3 the inference pipeline, and Figure 4 the network.

### 3.2. Feature Representation: Multi-Lag Autocorrelation

Given the snapshots ${\left\{x\left(t\right)\right\}}_{t=1}^{T}$, we form short-range temporal autocorrelations up to lag τ,

(13) $${\hat{R}}_{x}\left[\ell \right]=\frac{1}{T-\ell }\sum_{t=\ell +1}^{T}x\left(t\right)\;x^{H}\left(t-\ell \right)\in C^{N\times N},\;\ell =0,\ldots ,\tau -1,$$

stack the τ complex matrices along a channel axis, and split the real and imaginary parts,

(14) $${\mathrm{Stack}}_{ℜℑ}:\;Z\mapsto \left[\begin{array}{c} ℜ\{Z\}\\ ℑ\{Z\} \end{array}\right]\in R^{2N\times N},$$

so that the network input has shape B×τ×2N×N for a batch of *B* scenes. Second-order statistics contain exactly the information that subspace methods use while averaging out temporal noise, and the autocorrelation removes absolute phase and time shifts.

Contribution of non-zero lags

The zero-lag matrix ${\hat{R}}_{x}\left[0\right]$ is the sample covariance; under coherent multipath, its signal part collapses to rank one (8). The non-zero lags carry two kinds of information that are not provided by the zero lag: first, for white noise, $E\{n\left(t\right)n^{H}\left(t-\ell \right)\}=0$ for ℓ≠0, so the noise term vanishes and the signal structure is exposed without a noise floor; second, and more important under coherence, the direct and cross terms acquire different temporal weights. Repeating the derivation of (7) at lag *ℓ* gives

(15) $$\begin{array}{cc} R_{x}\left[\ell \right] & =P\;r\left(\ell \right)\;aa^{H}+{\left|g\right|}^{2}P\;r\left(\ell \right)\;bb^{H}\\ \; & \;+g\;P\;r\left(\ell -\tau \right)\;ab^{H}+g^{*}P\;r\left(\ell +\tau \right)\;ba^{H}+\sigma_{n}^{2}\delta \left[\ell \right]\;I_{N}. \end{array}$$

At ℓ=0, the four signal terms are weighted by $\left(1,1,\gamma ,\gamma^{*}\right)$ with |γ|≈1, and combine into the single outer product (8). At ℓ≠0, they are weighted by (r(ℓ),r(ℓ),r(ℓ−τ),r(ℓ+τ)). Whenever r(ℓ−τ)≠r(ℓ+τ), which holds for every τ≠0 because the autocorrelation of a band-limited source is not periodic, the terms no longer factorize, and the stacked matrices ${\left\{R_{x}\left[\ell \right]\right\}}_{\ell }$ jointly have rank two. Therefore, the separation between a and b can be recovered from the lag stack, although not from the sample covariance alone, and the second singular value of the stack grows with |r(ℓ−τ)−r(ℓ+τ)|. This argument also shows the limit of the representation; the information in the non-zero lags is carried by the source autocorrelation across the lag span, so it depends on the temporal statistics of the sources (Section 5.7). In our corpus, the source autocorrelation decays from 0.99 at lag 1 to 0.81 at lag 7, consistent with sinc(0.05ℓ) for a bandwidth of 5% of the sampling rate (10% of the Nyquist band). Therefore, τ=8 lags span the informative range without inflating the input. A full eigengap analysis for K>1 direct sources and multiple replicas is left for future work.

### 3.3. Network Architecture

A compact two-dimensional U-Net processes the lag–sensor grid with three encoder/decoder levels (Figure 4). Each encoder stage applies two 3×3 convolutions with normalization and nonlinearity, followed by 2×2 downsampling; the decoder mirrors this with transposed convolutions and skip connections. The lag–sensor tensor can be viewed as an image, with its textures being the algebraic regularities of an array covariance; the main diagonal encodes the per-sensor power, the near-diagonals encode short-baseline correlations, and longer-range structure encodes coupling and cross-lag correlations. The growing receptive field of the encoder aggregates the context across both lags (coherence and rank structure) and sensors (array-wide distortions). The skip connections preserve the fine conjugate-symmetric patterns that downsampling would otherwise wash out and on which accurate eigengaps depend. The network has 323,070 trainable parameters (Section 5.9) and is translation-equivariant along both axes.

The nonlinearity is an anti-rectifier, AR(X)=[ReLU(X)∥ReLU(−X)] (concatenation along channels), which keeps the sign information carried by the real/imaginary split. This is a design convenience rather than a contribution; in the ablation of Section 5.5, plain ReLU provides comparable performance.

### 3.4. Covariance Head: Hermitian Projection and Diagonal Loading

The decoder predicts an N×N complex matrix through the inverse of (14). We enforce Hermitian symmetry and stabilize the conditioning

(16) $${\hat{R}}_{\mathrm{Herm}}=\frac{1}{2}\left({\hat{R}}_{\mathrm{raw}}+{\hat{R}}_{\mathrm{raw}}^{H}\right),\;\hat{R}={\hat{R}}_{\mathrm{Herm}}+\varepsilon \;I_{N},$$

with a small load ε>0. The projection maps the output onto the admissible set of array covariances, and Tikhonov-style loading keeps it positive definite and stabilizes the eigendecomposition performed by the downstream estimator. This matrix is the network output.

### 3.5. Training Data and Objective

Inputs

For each scene, we synthesize snapshots under the imperfect array of Section 2.3, with per-scene randomization of the gain/phase errors G, position jitter $\left\{\varepsilon_{n}\right\}$, and coupling M together with optional coherent replicas as in Section 2.2 and additive noise at the target SNR. The corrupted snapshots are converted into the lag stack of Section 3.2.

Target

Supervision is provided by a clean covariance on the nominal manifold of Section 2.1, with no imperfections and no noise. With $A_{0}\left(\theta \right)$ the nominal steering matrix of the *K* direct sources and $R_{s}^{★}⪰0$ the source covariance, the target is

(17) $$R^{★}=A_{0}\left(\theta \right)\;R_{s}^{★}\;A_{0}^{H}\left(\theta \right),$$

in which the leading *K* eigenvectors span the signal subspace of the direct sources. The target is a closed-form object that exists for any scene once the geometry is specified; no angle label enters the loss.

Objective

The network is trained with the normalized reconstruction loss

(18) $$L_{\mathrm{rec}}=\frac{1}{N^{2}}∥\hat{R}-R^{★}{∥}_{F}^{2}$$

and nothing else. Training and inference are summarized in Algorithms 1 and 2. Subspace alignment, dominant eigenvector, and eigenvalue losses on predicted eigenpairs were tested in the ReconUNet-EVD variant (Appendix A). Section 5.5 shows that they do not improve the angular accuracy delivered to the estimator, and degrade it under an equal training budget.
**Algorithm 1:** Training
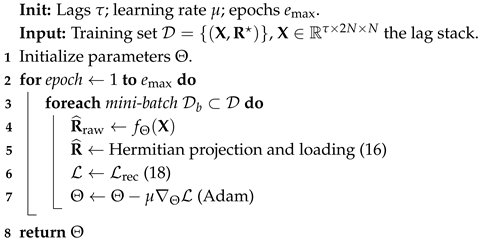


**Algorithm 2:** Inference
   **Init**: Trained Θ; known *K*; classical estimator G.
   **Input**: Snapshots X.
**_1_** Form the lag stack via (13)–(14).
**_2_** $\hat{R}\leftarrow f_{\Theta }\left(\;\cdot \;\right)$ followed by (16)
**_3_** $\hat{\theta }\leftarrow G\left(\hat{R};K\right)$    (the estimator is unmodified and performs its own EVD)
**_4_** **return** $\hat{\theta }$

What the network can and cannot learn about the array

The target $R^{★}$ lies on the nominal manifold, whereas the input carries a per-scene random hardware operator H=MG and random sensor positions; the network cannot identify H from a single scene. Therefore, minimizing (18) over the corpus drives the network toward the conditional expectation of the clean covariance given the corrupted lag stack, $E\{R^{★}|X\}$, under the training distribution of imperfections, multipath configurations, SNR, and source statistics. In this sense, ReconUNet is a learned and data-driven covariance regularizer, closer to a minimum mean square error estimator under a prior than to a calibration procedure. Three consequences follow, which are measured in Section 5. First, the network cannot remove the part of the error that is specific to a single array realization. On clean single-source scenes, the residual floor is set by the prior; here, a classical estimator on the raw sample covariance is slightly more precise. Second, the network can recover what is common to the training distribution and visible in the input. Coupling and gain/phase errors are common to all lags, whereas the source correlation structure varies across lags (15); therefore, the lag stack identifies the signal subspace much better than it identifies the manifold distortion. This explains why the gains are concentrated in coherent and crowded scenes, as the subspace is the problem rather than the manifold; it also explains why a network trained on a single fixed imperfection realization has the same performance as one trained with per-scene randomization (Section 5.5). Third, the network inherits every assumption of its training distribution, including the temporal correlation of the sources, which determines the content of the non-zero lags; Section 5.7 measures this dependence. Thus, ReconUNet is complementary to array calibration, not a substitute for it. When calibration data are available, a calibrated manifold reduces the mismatch that the network has to absorb.

### 3.6. Inference and Classical Integration

At test time, we compute the lag stack, apply the network once, and pass $\hat{R}$ to the chosen estimator in place of the sample covariance (Algorithm 2). Nothing in the estimator changes. Bartlett, MVDR, and MUSIC scan their spectra, Root-MUSIC roots its polynomial, and ESPRIT and Unitary-ESPRIT solve their invariance equations, all on $\hat{R}$. The number of sources *K* is supplied to the estimator exactly as it would be for the sample covariance.

## 4. Experimental Setup

This section provides the data synthesis, imperfection presets, scenarios, ReconUNet training protocols and its variants, learned and classical baselines, and metrics that are required to reproduce the results. All experiments are simulations. The code, data generation scripts, evaluation harness, and trained weights are available at https://github.com/itamaralmog96/ReconUNet-DoA (accessed on 1 November 2026).

Array and signals

We use a ULA with N=8 elements at half-wavelength spacing, a carrier frequency of 2.45 GHz, and T=512 snapshots per scene. Sources are complex Gaussian processes band-limited to 5% of the sampling rate (10% of the Nyquist band), which gives a coherence time of about 20 snapshots. Replica delays are fractional, and are applied exactly through an FFT phase ramp. The SNR is defined with respect to the main source power *P* as $10\log_{10}\left(P/\sigma_{n}^{2}\right)$. Direct-path DoAs are drawn uniformly on $\left[30^{\circ },150^{\circ }\right]$ with a minimum separation of $10^{\circ }$ between direct sources; this avoids the endfire region, where the ULA manifold is least informative. Replica angles are unconstrained.

Imperfection presets

We use two presets of the model in Section 2.3, each drawn independently per scene. The *mild* preset has $g_{n}∼U\left[10^{-0.1/20},10^{0.1/20}\right]$ (±0.1 dB), $\delta_{\phi }=1^{\circ }$, coupling magnitude ρ=0.02 with $\varphi_{c}=-100^{\circ }$ and δ=0.9 ($v_{k}∼U\left[0.55,1.45\right]$), and $\sigma_{\mathrm{pos}}=0.01\lambda$ (1% of the wavelength, isotropic in the plane). The *harsh* preset multiplies each magnitude by five: ±0.5 dB, $\delta_{\phi }=5^{\circ }$, ρ=0.1, and $\sigma_{\mathrm{pos}}=0.05\lambda$. All learned models are trained on the mild preset only; the harsh preset is a distribution shift introduced at test time.

Scenarios

In addition to the mixed test split described below, four scenarios of 1000 scenes each are evaluated across SNR: *Basic* (one direct source, no multipath); *Moderate* (two direct sources plus one coherent replica of one of them); *Crowded* (four direct sources plus three coherent replicas spread across the emitters); and *Saturated* (one direct source plus six coherent replicas, i.e., seven coherent arrivals on eight elements). The saturated configuration lies inside the training distribution, since single-source training scenes carry up to six replicas, but is the heaviest multipath load that the array can carry and is included as a stress test. In every scenario, *K* is the number of direct sources, and is supplied to every method; the scene geometry is fixed across SNR levels and only the noise power changes, making the SNR sweeps directly comparable.

Training corpus and splits

The corpus contains $2\times 10^{6}$ training, 50,000 validation, and 50,000 test scenes (corrupted input and clean target), with K∈{1,2,3,4} direct sources of equal power drawn uniformly, M∼U{0,…,7−K} coherent replicas, SNR drawn uniformly in [−10,10] dB, mild imperfections randomized per scene, and a fixed master seed. Every scene is rendered deterministically from a 96-byte manifest row, so the corpus is reproducible without storing waveforms. The held-out test split used in Section 5.3 contains 12,000 scenes (3000 per value of *K*) drawn from the test partition with the same SNR and imperfection distribution as in training. Of its scenes, 81% carry coherent replicas; single-source scenes carry up to six, making K=1 the bucket with the heaviest multipath.

ReconUNet training

We use lag depth τ=8, anti-rectifier activation, dropout, diagonal load ε=1, and loss (18) only. Optimization uses Adam with learning rate $10^{-4}$ and weight decay $10^{-5}$, batch size 2048, gradient clipping at unit $\ell_{2}$ norm, and fp32 arithmetic, for up to 300 epochs with early stopping (patience 25) and ReduceLROnPlateau (factor 0.7, patience 5) on the validation loss. The model has 323,070 trainable parameters. It ran 207 epochs in 8.9 h on a single NVIDIA RTX 2000 Ada Generation GPU (NVIDIA Corporation, Santa Clara, CA, USA) and selected epoch 182 on validation loss, with a validation angular error of $1.33^{\circ }$.

Variants

Two variants share the backbone, data interface, and optimizer settings. ReconUNet-EVD adds the eigendecomposition heads and the four-term loss of Appendix A (337,842 parameters). This was the model used in the earliest version of this paper; it ran 109 epochs in 4.8 h and selected epoch 84 (validation angular error $2.17^{\circ }$). ReconUNet-BW is the proposed network trained on a corpus in which the source bandwidth is drawn per scene: log-uniformly between 1% and 40% of the sampling rate with probability 0.85, and white otherwise. Replica delays are drawn from the same distribution as in the main corpus. Every other property of the corpus and of training is unchanged, and the model is trained from scratch. It ran 204 epochs in 8.8 h and selected epoch 179 (validation angular error $1.46^{\circ }$ on the randomized-bandwidth validation split). This is the network used in Section 5.7.

Learned baselines

Three learned frontends were trained on the same corpus using the same SNR range, optimizer, batch size, scheduler, early-stopping rule, and epoch budget as ReconUNet, and were evaluated through the same code path on the same held-out scenes. SubspaceNet [25] uses the official implementation, adapted to our data interface and trained through its differentiable Root-MUSIC on angle labels. DA-MUSIC [26] has a fixed-width head; following its published protocol, one instance was trained per source count on that count’s 500,000-scene subset (15,187 parameters each). The instances for K≥2 reached the 300-epoch budget without early stopping, and were continued to a 600-epoch cap (best epochs 466, 595, and 557); all DA-MUSIC results below use the continued models. SubViT is a covariance-input vision transformer regressor [34] that represents the direct prediction approach [30]. It has embedding dimension 768, depth 6, and 42.6M parameters, is trained with the grid-BCE spectrum objective of that approach, and is read out by picking the *K* strongest local maxima. Deviations from the published baseline code (batched MUSIC spectrum, Hermitian PSD surrogate with a shared robust eigendecomposition, and time-major GRU input) are documented in the repository and do not change any trainable parameters.

Classical baselines

We use the Bartlett and MVDR beamformers, MUSIC on a $1^{\circ }$ grid over $\left[30^{\circ },150^{\circ }\right]$ with three-point parabolic peak refinement and local maximum selection, Root-MUSIC, ESPRIT, and Unitary-ESPRIT, which incorporates forward–backward averaging. All use fp64 eigendecompositions, and each is evaluated both on the raw sample covariance and on the covariance reconstructed by ReconUNet. As the classical remedy for coherence, we also evaluate forward–backward spatial smoothing (FBSS) [13] with sub-array lengths L∈{5,6,7}, followed by Root-MUSIC.

Metrics

The primary metric is the pooled RMSE, which is the square root of the mean of all squared per-source errors after optimal assignment (12) over a set of scenes. It is dominated by the tail of the error distribution. We also report the median per-scene RMSE, which describes the typical scene, and the percentile-bootstrap 95% confidence intervals (1000 resamples over scenes). The intervals are given in the result tables of Section 5 and in the text for the central comparisons, while the complete tables are provided in the repository. A uniformly distributed error over the $120^{\circ }$ sector has an RMSE of $34.6^{\circ }$; values in this range indicate threshold region breakdown. Where informative, we overlay the stochastic CRLB for a single source.

Use of generative AI tools

Generative AI tools were used only as writing aids during the preparation of this manuscript and its revisions, not for the research itself. The large language models ChatGPT (GPT-5, OpenAI, San Francisco, CA, USA) and Claude (Claude Opus 5.5, Anthropic, San Francisco, CA, USA) were used to help draft and rephrase parts of the text, to condense the abstract to the journal’s word limit, and for language editing (grammar, spelling, punctuation, and formatting). No generative AI tools contributed to the conception or design of the study, the signal, multipath, and array imperfection models, the design of the ReconUNet architecture or its loss function, the implementation of the software, the generation, processing, or analysis of the data, the production of the figures and tables, or the interpretation of the results. The authors reviewed and edited all AI text and take full responsibility for the content of this publication.

## 5. Results and Discussion

We now proceed from the sanity check to stress tests consisting of classical estimators under matched conditions (Section 5.1), classical versus aided estimators under coherent multipath and imperfections (Section 5.2), a comparison with learned frontends on the held-out split (Section 5.3) and across SNR in the four scenarios (Section 5.4), and the ablation that led to the configuration proposed in this paper (Section 5.5), followed by snapshots and source separation (Section 5.6), source bandwidth (Section 5.7), robustness and limitations (Section 5.8), and computational cost (Section 5.9). Unless stated otherwise, “ReconUNet” denotes the proposed covariance-only network with the Root-MUSIC backend.

### 5.1. Classical Estimators Under Matched Conditions

**Figure 5:**
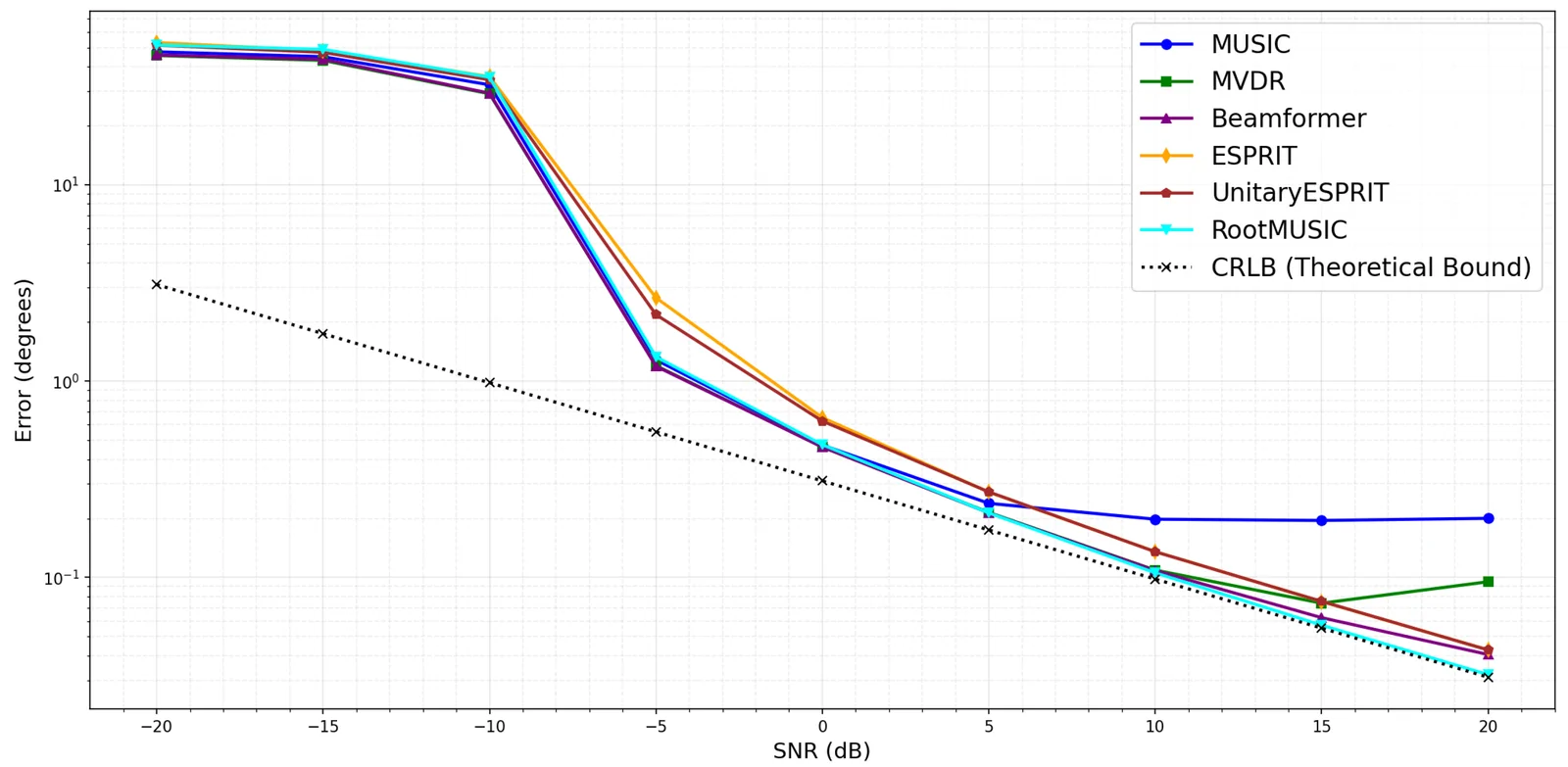
Classical estimators on a perfect array, Basic scenario, $1^{\circ }$ scan grid: RMSE vs. SNR with the CRLB.

Before introducing the frontend, we verify the classical implementations in the Basic scenario with a perfect array. Figure 5 and Table 2 show that Root-MUSIC, ESPRIT, and Unitary-ESPRIT track the CRLB closely, whereas the grid-scanning methods flatten at high SNR at the level set by the $1^{\circ }$ grid with parabolic refinement (a $0.1^{\circ }$ grid removes this floor at ten times the scan cost). At −10 dB, all estimators are in the threshold region, and their ordering is not meaningful. One detail that recurs in every table is that at high SNR, grid MUSIC with parabolic refinement has a pooled RMSE several times that of Root-MUSIC ($0.197^{\circ }$ against $0.032^{\circ }$ here), although its median error is similar ($0.19^{\circ }$ against $0.15^{\circ }$ at 20 dB on an imperfect array). We verified the implementation, finding that the gap comes from the 0.6% of scenes in which the refined peak selection returns a spurious local maximum and that the squared errors of these few scenes dominate the pooled statistic. For this reason, we report medians alongside pooled values throughout; the check confirms that classical subspace processing is near-optimal when its assumptions hold. The task of the frontend is to restore this behavior when the assumptions do not hold.

### 5.2. Coherent Multipath and Array Imperfections: Raw Versus Reconstructed Covariance

Table 3 reports every classical estimator at 0 dB in the four scenarios on the raw sample covariance and the ReconUNet covariance, under both the mild and harsh imperfection presets. Three observations follow. First, coherent multipath is the dominant failure mode of classical subspace methods. In the Moderate scenario, Root-MUSIC stays at $5.91^{\circ }$ and does not improve with SNR (Section 5.4), which is the signature of the rank collapse described in Section 2.2. The reconstructed covariance brings this to $0.61^{\circ }$ (95% interval ${\left[0.56,0.65\right]}^{\circ }$, against ${\left[3.8,7.7\right]}^{\circ }$ raw), and in the Crowded scenario from $8.87^{\circ }$[8.0,9.7] to $2.45^{\circ }$[1.8,3.1]. Second, the effect does not depend on the estimator. The same weights serve Root-MUSIC, ESPRIT, and Unitary-ESPRIT with almost identical results ($0.61^{\circ }$ for all three in the Moderate scenario) and also improve grid MUSIC, for which the remaining gap is due to its spurious peak outliers. Third, the frontend degrades gracefully under the harsh preset (Moderate: $0.61^{\circ }\to 2.14^{\circ }$; Crowded: $2.45^{\circ }\to 4.15^{\circ }$) even though it was trained on mild imperfections only. The ReconUNet-EVD variant is worse than the proposed network in every column of both presets; in the Basic scenario at 0 dB, with a single clean source, raw Root-MUSIC remains marginally more precise ($0.25^{\circ }$, interval [0.24,0.26], against $0.29^{\circ }$, [0.27,0.30]), for the reason given in Section 3.5. This gap is much smaller than for the ReconUNet-EVD variant ($0.37^{\circ }$), and both are within $0.03^{\circ }$ under the harsh preset.

Comparison with spatial smoothing

Forward–backward spatial smoothing is the standard classical remedy for coherent sources, and Table 3 includes it with sub-array lengths of 5, 6, and 7. On this eight-element array, it does not help; FBSS with L=7 gives $6.31^{\circ }$ in the Moderate scenario and $9.61^{\circ }$ in the Crowded scenario, against $5.91^{\circ }$ and $8.87^{\circ }$ for Root-MUSIC on the unsmoothed covariance and $0.61^{\circ }$ and $2.45^{\circ }$ with the reconstructed covariance, while shorter sub-arrays are worse still. When the number of coherent arrivals approaches the number of elements, the aperture lost to smoothing costs more than the decorrelation gains. This is the regime in which a learned frontend has something to offer.

### 5.3. Comparison with Learned Frontends on the Held-Out Split

**Figure 6:**
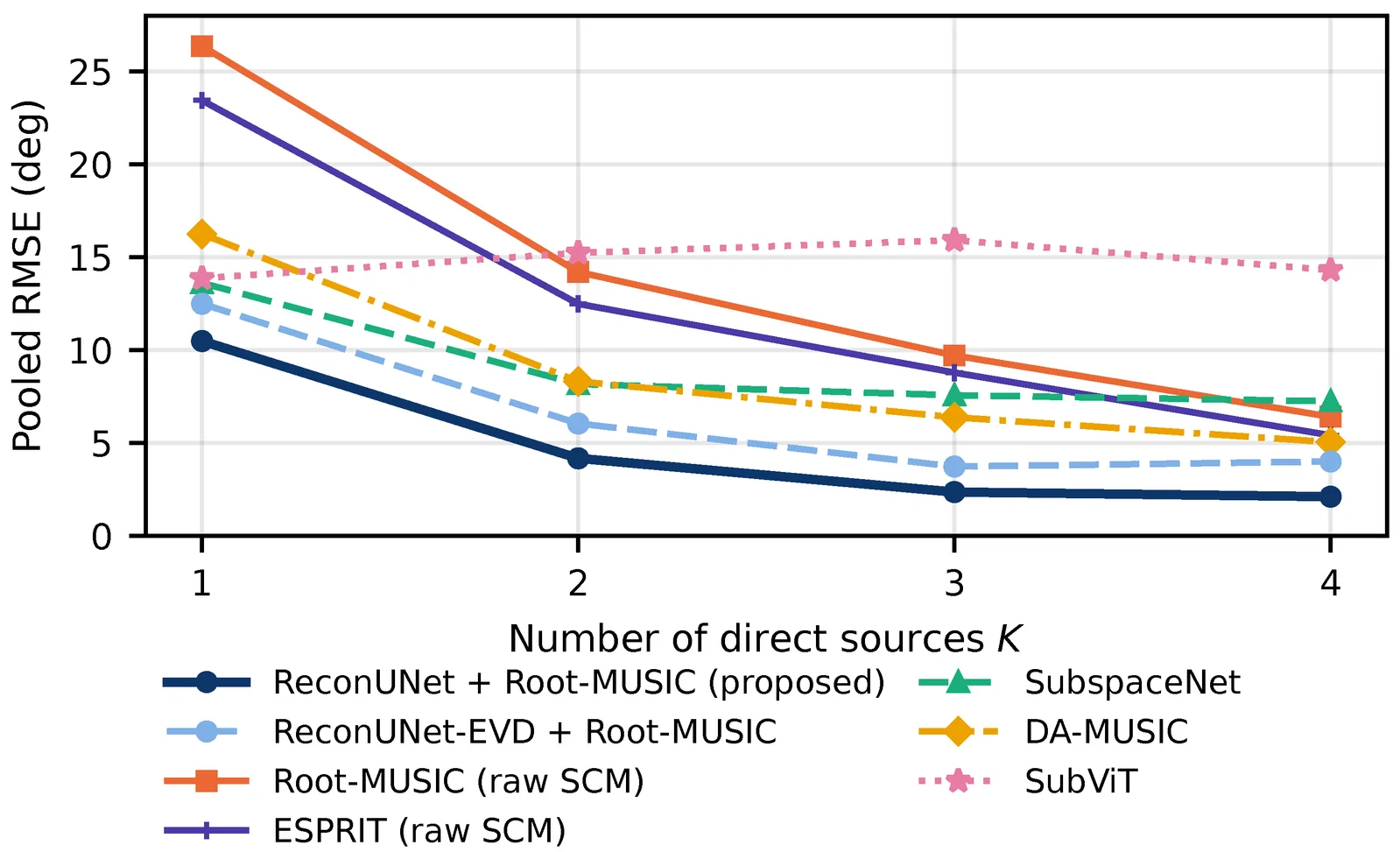
Pooled RMSE versus number of direct sources on the held-out split. The decrease with *K* common to all methods reflects the composition of the corpus (coherent replicas drawn from {0,…,7−K}, so K=1 scenes carry the most multipath), not an easier estimation problem; methods are compared within each *K*.

Table 4 and Figure 6 compare ReconUNet with SubspaceNet, DA-MUSIC, SubViT, the ReconUNet-EVD variant, and the two best classical estimators on the 12,000-scene test split, by number of sources and pooled RMSE. On the pooled metric, ReconUNet is best overall: $4.23^{\circ }$ (95% interval [3.76,4.67]), against $5.79^{\circ }$[5.35,6.18] for ReconUNet-EVD, $7.91^{\circ }$[7.58,8.25] for DA-MUSIC, $8.37^{\circ }$[8.02,8.70] for SubspaceNet, and $12.43^{\circ }$[11.96,12.88] for raw Root-MUSIC. It is also best for every number of sources, with intervals that do not overlap those of any baseline for K≥2. The pooled error of every method decreases with *K*. This is not because more sources are easier to estimate; rather, it is a property of the corpus, in which the number of coherent replicas is drawn uniformly from {0,…,7−K}. Single-source scenes carry three replicas on average, while four-source scenes carry one and a half; because coherent replicas are the dominant cause of large errors, the K=1 bucket is the hardest. Thus, the comparisons should be read within the rows, not along the columns. Within each row, the advantage of ReconUNet over the best baseline grows as the replica load falls and the number of direct sources rises, from $10.5^{\circ }$ against $12.5^{\circ }$ at K=1 to $2.1^{\circ }$ against $4.0^{\circ }$ at K=4.

**Figure 7:**
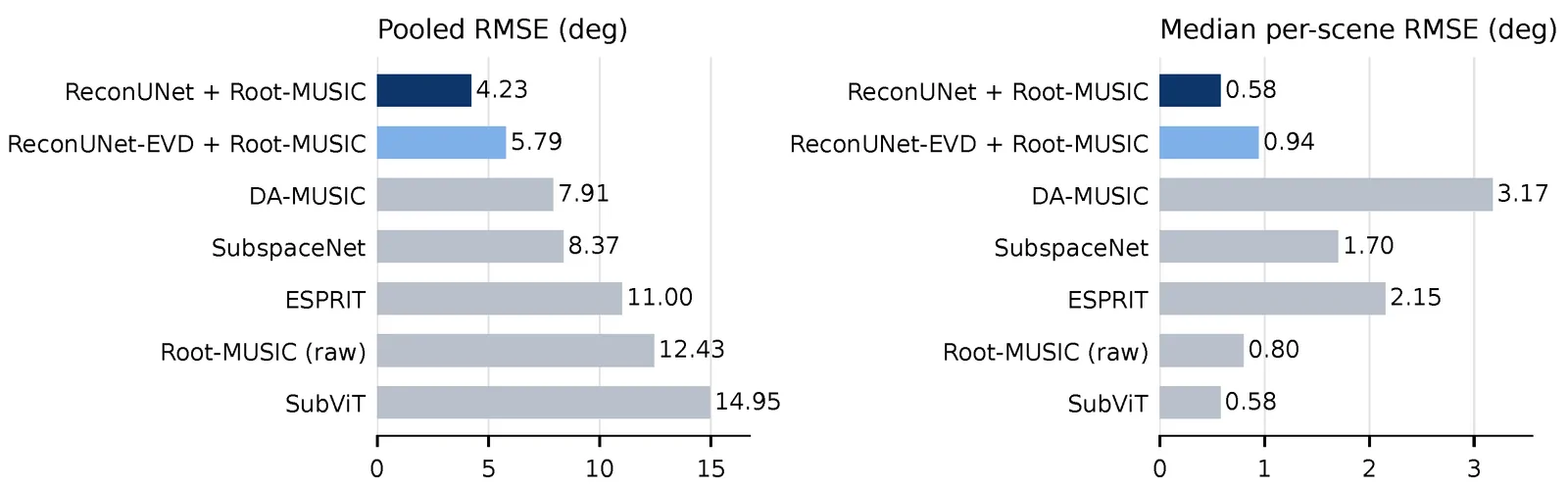
Held-out split, all *K*: pooled RMSE (**left**) and median per-scene RMSE (**right**). The two panels rank the baselines differently; ReconUNet is best on the pooled metric and tied for best on the median metric.

The median column shows what the pooled metric hides (Figure 7). Raw Root-MUSIC is precise on the typical scene ($0.80^{\circ }$) but fails badly on a minority of coherent scenes that dominate its pooled error. SubViT has a good median ($0.58^{\circ }$) along with the worst pooled error ($14.95^{\circ }$). ReconUNet has both the lowest pooled error and a median of $0.58^{\circ }$, equal to that of SubViT and lower than that of raw Root-MUSIC. On this test split, it removes the catastrophic failures that coherent multipath and crowding cause in subspace estimators without giving up precision on the typical scene. In contrast, the ReconUNet-EVD variant has a median ($0.94^{\circ }$) worse than that of raw Root-MUSIC; the precision loss reported for the first version of this method was caused by the eigendecomposition heads and the composite loss, not by covariance reconstruction.

### 5.4. Scenario Study Across SNRs

**Figure 8:**
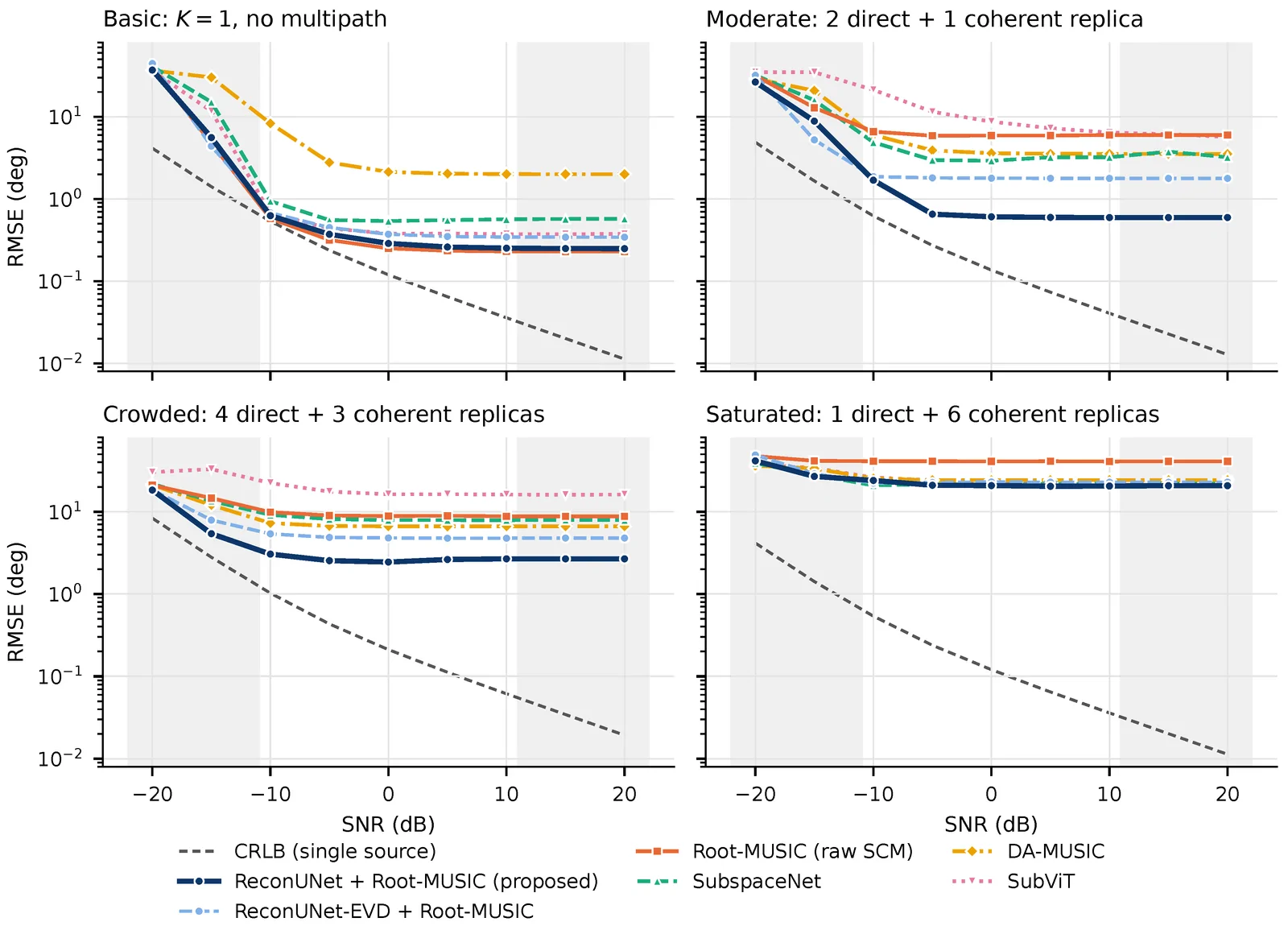
Pooled RMSE versus SNR (1000 scenes per point, mild imperfections) in the four scenarios, shown on a common logarithmic scale. Learned methods were trained on [−10,10] dB; the shaded bands lie outside that range for all of them. Thin dashed blue: the ReconUNet-EVD variant; dashed gray line: single-source stochastic CRLB.

Figure 8 plots the pooled RMSE against the SNR for the four scenarios and six methods, together with the single-source CRLB; the shaded bands mark SNRs outside the [−10,10] dB training range, where every learned method extrapolates. Four regimes are visible. In the threshold region (−20 and −15 dB), all estimators approach the $\approx 35^{\circ }$ error of uniform guessing, and their ordering is not meaningful. In the Basic scenario, all methods converge quickly. Above 0 dB, raw Root-MUSIC ($0.23^{\circ }$ at 20 dB) and ReconUNet ($0.25^{\circ }$) are nearly equal and better than the other learned methods, for which the floors lie between $0.34^{\circ }$ (ReconUNet-EVD) and $2.0^{\circ }$ (DA-MUSIC). The floors do not move between 10 and 20 dB, so they are set by the learned prior (Section 3.5) rather than by extrapolation beyond the training SNR. In the Moderate and Crowded scenarios, coherent multipath makes the classical curves flat from −5 dB upward, and ReconUNet is best at every SNR from −10 dB upward. At 0 dB, it attains $0.61^{\circ }$ against $5.91^{\circ }$ for Root-MUSIC and $2.92^{\circ }$ for SubspaceNet in the Moderate scenario and $2.45^{\circ }$ against $8.87^{\circ }$ and $6.65^{\circ }$ for DA-MUSIC in the Crowded scenario. In the Saturated scenario, seven coherent arrivals on eight elements reach the degrees-of-freedom limit of the array. Raw Root-MUSIC fails on most scenes ($41^{\circ }$ pooled), and all learned methods lie between $21^{\circ }$ and $24^{\circ }$ pooled, with ReconUNet the lowest ($20.8^{\circ }$). The medians are informative here; aided Root-MUSIC resolves the typical scene ($1.2^{\circ }$ median, against $3.9^{\circ }$ raw), so the pooled figure reflects a fraction of unrecoverable scenes rather than uniform failure. No method recovers those scenes.

Qualitative diagnostics

**Figure 9:**
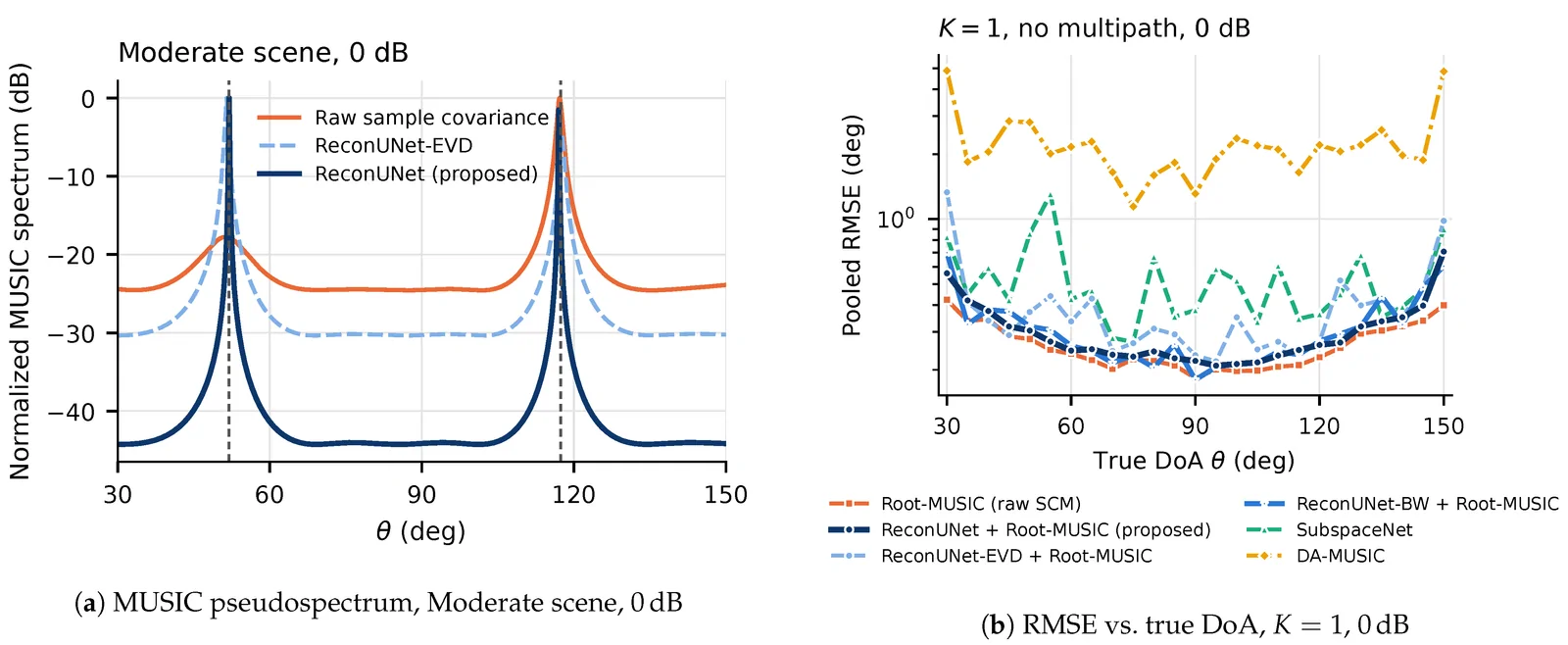
Qualitative diagnostics. (**a**) Normalized MUSIC pseudospectra from the raw sample covariance and from the covariances reconstructed by ReconUNet and ReconUNet-EVD for a Moderate scene (K=2 plus one coherent replica); dashed lines mark the true DoAs. (**b**) Pooled RMSE as a function of the true direction for a single source without multipath (200 scenes per angle, mild imperfections, 0 dB).

Figure 9 shows the effect of the reconstruction on the statistic that a subspace estimator uses. In a Moderate scene at 0 dB, the MUSIC pseudospectrum computed from the raw sample covariance has one of its two main lobes suppressed by about 18 dB, the signature of the rank collapse described in Section 2.2. The pseudo-spectrum computed from the reconstructed covariance has two sharp peaks at the true directions, within 1.5 dB of each other, and a noise floor about 20 dB below that of the raw spectrum. The ReconUNet-EVD variant recovers the second peak less completely (−4.6 dB), and its floor is 14 dB higher. The second panel sweeps a single source without multipath across the sector, a case in which nothing is broken for the classical estimator. All methods degrade toward the edges of the sector, where the ULA manifold flattens. Raw Root-MUSIC is the most precise ($0.27^{\circ }$ pooled over all directions), and ReconUNet and ReconUNet-BW follow closely ($0.33^{\circ }$ and $0.34^{\circ }$; at broadside, $0.22^{\circ }$ and $0.18^{\circ }$ against $0.18^{\circ }$). ReconUNet-EVD ($0.47^{\circ }$), SubspaceNet ($0.58^{\circ }$), and DA-MUSIC ($2.4^{\circ }$) are less accurate across the whole sector.

### 5.5. Ablation and the Choice of Architecture

To isolate the design choices, Table 5 retrains variants of the ReconUNet-EVD model of the first version of this paper under a reduced but identical protocol: a fixed and seeded 10% subset of the training corpus (and of the validation split used for checkpoint selection), 40 epochs, and all other settings as in Section 4. Each run takes 15–19 min. Each variant is evaluated on the test split and on the Moderate and Crowded scenarios at 0 dB with the Root-MUSIC backend. In addition to the angular error, we report the relative covariance reconstruction error ${∥\hat{R}-R^{★}∥}_{F}/{∥R^{★}∥}_{F}$, the projection-Frobenius distance between the leading-*K* subspaces of $\hat{R}$ and $R^{★}$, and the relative eigengap error, showing the effect of each component on the statistics.

The outcome is unambiguous, and not what we expected when we designed the first version of the method. What matters is the covariance reconstruction objective on the nominal manifold target and the multi-lag input. Removing the EVD heads altogether and training the U-Net on $L_{\mathrm{rec}}$ alone gives the best variant on every statistic except the eigengap error: $6.05^{\circ }$ pooled RMSE against $9.22^{\circ }$ for the reference, with the lowest covariance error and the smallest subspace distance. Among the loss terms, $L_{\mathrm{eig}}$ is harmful under this budget; removing it improves the test split from $9.22^{\circ }$ to $7.37^{\circ }$ and the Moderate scenario from $4.05^{\circ }$ to $1.85^{\circ }$, while $L_{\mathrm{dom}}$ has no measurable effect and $L_{\mathrm{proj}}$ adds a small improvement on top of $L_{\mathrm{rec}}$ ($7.27^{\circ }\to 7.03^{\circ }$) that is lost in the full composite loss. The training logs explain the mechanism: with unit weights, the eigenvalue term dominates the composite objective (the validation loss of the reference is ≈21, against ≈2.3 without $L_{\mathrm{eig}}$), so the network spends most of its capacity and gradient on the spectrum rather than on the covariance that the estimator uses, and converges much more slowly. The anti-rectifier is not decisive either, with plain ReLU reaching $8.54^{\circ }$ against $9.22^{\circ }$. The multi-lag input helps, as the single-lag variant is the worst on the test split ($9.87^{\circ }$) and by far the worst in the Moderate scenario ($6.08^{\circ }$ against $4.05^{\circ }$); this is consistent with the analysis in Section 3.2.

Therefore, we adopted the covariance-only configuration as the proposed method and carried out full-scale training using the protocol from Section 4. The full-scale results confirm the ablation. The covariance-only network reaches a validation angular error of $1.33^{\circ }$, against $2.17^{\circ }$ for ReconUNet-EVD. It improves the pooled test split error from $5.79^{\circ }$ to $4.23^{\circ }$ and the median from $0.94^{\circ }$ to $0.58^{\circ }$ (Table 4). It is better in every scenario and imperfection preset of Table 3 while being faster and having fewer parameters (Section 5.9). It needs more epochs to converge (207 against 109) because it continues to improve long after the composite loss of ReconUNet-EVD has plateaued.

Two further rows of Table 5 answer several questions raised earlier. Training on one fixed imperfection realization instead of per-scene randomization changes nothing: $9.27^{\circ }$ against $9.22^{\circ }$ on the randomized test split and $9.24^{\circ }$ against $9.25^{\circ }$ on the test split rendered with the fixed realization. Therefore, at the mild imperfection level of this corpus, the network learns no realization-specific correction, as predicted by the argument of Section 3.5. Its tolerance of the harsh preset (Table 3) is a property of the regularized covariance as opposed to a calibration. Finally, in the ReconUNet-EVD model, using the predicted eigenbasis directly instead of the numerical EVD of $\hat{R}$ gives identical estimates for every scene, so the eigenvector head adds nothing over a numerical EVD of the reconstructed covariance.

### 5.6. Snapshots and Source Separation

**Figure 10:**
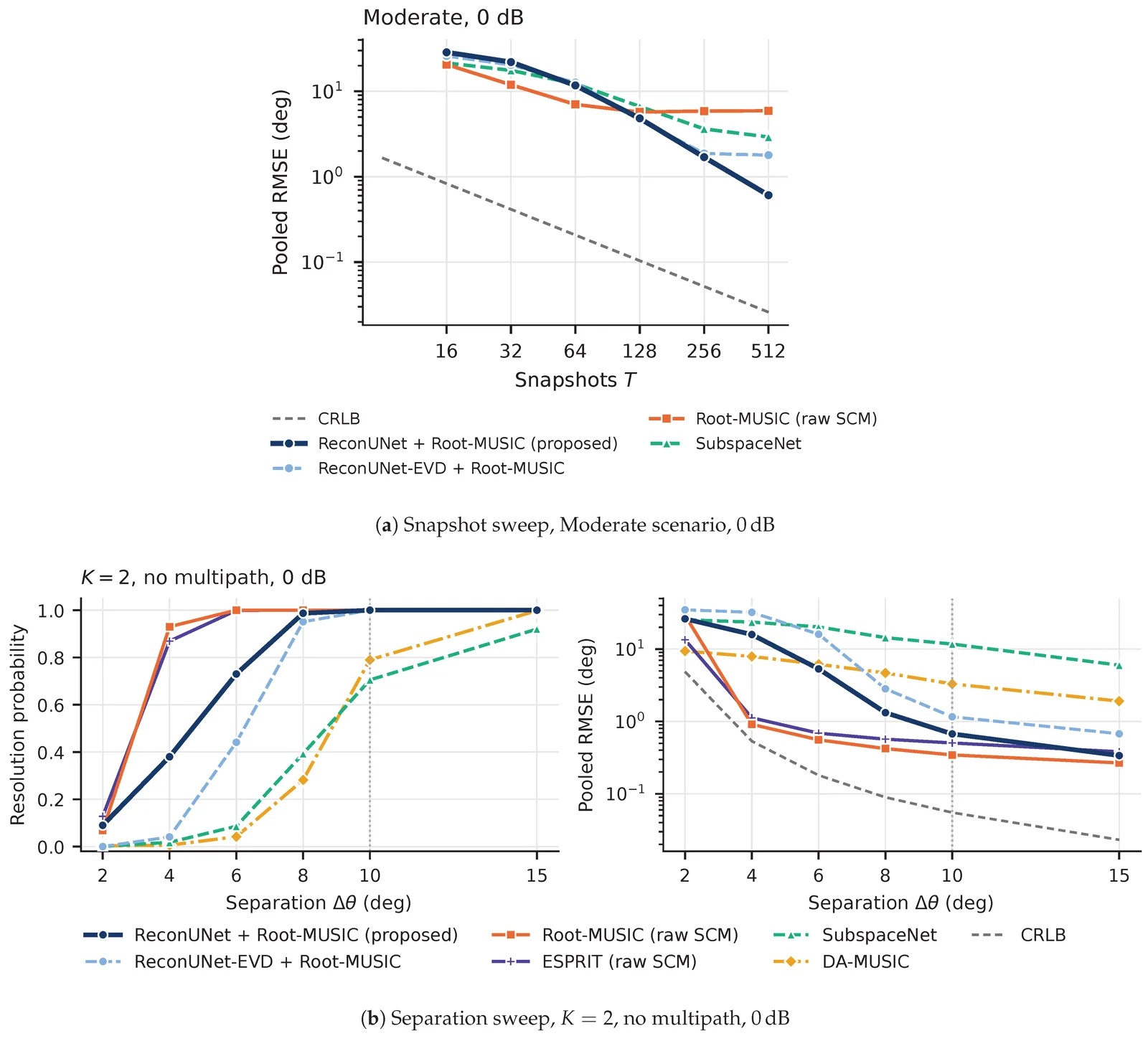
(**a**) Pooled RMSE versus number of snapshots for models trained at T=512 (1000 scenes per point). (**b**) Resolution probability (both sources within Δθ/2 of the truth) and pooled RMSE versus source separation; the dotted line marks the $10^{\circ }$ minimum separation of the training corpus.

Two further axes determine where the method can be used. Figure 10a sweeps the number of snapshots in the Moderate scenario at 0 dB, with the models trained at T=512 applied unchanged to contiguous windows of *T* snapshots. Raw Root-MUSIC already reaches its floor of about $6^{\circ }$ at T=64, and does not improve further because its error is set by the coherence-induced rank collapse rather than the estimation noise. ReconUNet is worse than raw Root-MUSIC below T≈100 ($22.0^{\circ }$ against $11.9^{\circ }$ at T=32; $11.7^{\circ }$ against $7.0^{\circ }$ at T=64), which is because the lag stack estimates that it receives are far noisier than anything seen during training. It crosses below raw Root-MUSIC at T=128 ($4.8^{\circ }$ against $5.7^{\circ }$), then keeps improving to $1.7^{\circ }$ at T=256 and $0.61^{\circ }$ at T=512. The ReconUNet-EVD variant and SubspaceNet both behave similarly. Therefore, the proposed frontend is not a remedy for snapshot starvation; instead, it removes the coherence floor, which cannot be removed simply by using more snapshots. It also assumes the sampling rate used in training. When applied to decimated snapshots, it fails at every *T*, as decimation changes the source autocorrelation at each lag, and consequently the input distribution. Section 5.7 examines the same effect through the source bandwidth.

Figure 10b sweeps the separation between two equal-power sources without multipath at 0 dB, a regime in which nothing is broken for the classical estimators, placing the learned methods at a disadvantage. Raw Root-MUSIC resolves the pair with probability 0.93 at $4^{\circ }$ and 1.0 from $6^{\circ }$. ReconUNet resolves it with probability 0.38 at $4^{\circ }$, 0.73 at $6^{\circ }$, 0.99 at $8^{\circ }$, and 1.0 from $10^{\circ }$ (the minimum separation in the training corpus). This is better than ReconUNet-EVD (0.04, 0.44, and 0.95) and much better than SubspaceNet and DA-MUSIC, but still short of the raw estimator; the results at −5 dB are similar. The learned covariance carries a prior for separations of at least $10^{\circ }$ and partially merges closer pairs. This method offers no super-resolution; as trained, it should not be used for sources closer than about $8^{\circ }$. Training with smaller separations is straightforward, and is left for future work.

### 5.7. Dependence on the Source Bandwidth

The analysis of Section 3.2 attributes the benefit of the non-zero lags to the temporal correlation of the sources, as a coherent replica becomes separable in the lag stack because r(ℓ−τ)≠r(ℓ+τ). This requires the source to stay correlated over the span of the stack. All models above were trained with one source spectrum, a bandwidth of 5% of the sampling rate, for which |r(ℓ)| decays only from 1 to 0.83 over the eight lags. Table 6 measures what happens when the test bandwidth differs. The test scenes have fixed geometry, imperfections, and replica delays; only the source spectrum changes, from 1% of the sampling rate to white. A second version in which the replica delays scale with the coherence time, as they would for physically longer paths, gives the same picture and is provided in the repository.

Three regimes appear for ReconUNet trained at 5%. For bandwidths up to 10% of the sampling rate, where the autocorrelation at the last lag is still above about 0.4, it remains better than every baseline in both scenarios, with its best results at and just below the training bandwidth. Narrower sources (B=0.01) are somewhat harder, since the replicas become more coherent and the lags more similar. For wider bandwidths, the source becomes decorrelated within the stack, the non-zero lags lose the structure that the network learned to read, and ReconUNet fails: $22.7^{\circ }$ at 20% and $30.9^{\circ }$ for white sources in the Moderate scenario, which is far worse than raw Root-MUSIC (5.3–$5.9^{\circ }$). The decorrelation also makes the replicas less coherent with the direct path, so both the classical estimators and DA-MUSIC (which reads the snapshots directly) improve with bandwidth. SubspaceNet, which also takes a lag stack as input, degrades in the same direction as ReconUNet, though less sharply. Therefore, a lag stack frontend trained at one bandwidth encodes that bandwidth.

To separate the effect of the training distribution from that of the representation, we trained ReconUNet-BW, the same network trained on bandwidths drawn between 1% and 40% of the sampling rate and on white sources (Section 4). Within the tested range, the failure disappears. ReconUNet-BW is the most accurate method in every row of Table 6 except the training bandwidth of the narrowband model. In the wideband rows, it reaches $0.53^{\circ }$ in the Moderate scenario and 1.8–$2.8^{\circ }$ in the Crowded scenario, against 5.3–$5.9^{\circ }$ and 7.6–$8.8^{\circ }$ for raw Root-MUSIC and 2.8–$3.1^{\circ }$ and 5.4–$5.8^{\circ }$ for DA-MUSIC. For white sources, the coherence problem itself weakens, as a delayed copy of a white waveform is uncorrelated with the direct path at lag 0 and appears instead at the lag equal to its delay; a network that has seen such inputs reads the replica from the lag stack in that regime as well. The same picture holds at −5 and +10 dB, in the variant with coupled replica delays, and in a third scenario with three direct sources and one replica (provided in the repository). Covering the wider range costs little at the nominal bandwidth. At 5%, ReconUNet-BW gives $0.78^{\circ }$ against $0.61^{\circ }$ in the Moderate scenario and $2.57^{\circ }$ against $2.45^{\circ }$ in the Crowded scenario; on the held-out split rendered at the nominal bandwidth, it gives $4.96^{\circ }$[4.51,5.38] against $4.23^{\circ }$, still well ahead of every baseline. Finally, we re-rendered the held-out split with each scene’s source at its own randomly drawn bandwidth, which models a test bandwidth that is unknown within the trained range. There, ReconUNet-BW attains $4.80^{\circ }$[4.36,5.23] (median $0.58^{\circ }$), against $7.65^{\circ }$ for DA-MUSIC, $11.60^{\circ }$ for raw Root-MUSIC, and $15.45^{\circ }$ for the narrowband ReconUNet. These results show that training over a range of bandwidths removes the failure observed for a network trained at a single bandwidth within the range covered by training. It does not remove the dependence of the representation on temporal correlation, which is intrinsic; the information in the non-zero lags is carried by the source autocorrelation across the lag span (Section 3.2). The training distribution changes only whether the network has learned how to read the lag stack under the temporal statistics that it encounters.

The consequence is a requirement imposed on the training data rather than on the network. The training corpus must cover the source bandwidths expected at test time, and more generally, the temporal statistics and sampling rate; when that range is known only roughly, training on a wide range is the safer choice, and incurs only a modest cost at the nominal bandwidth. We tested this only for the bandwidth and only in simulation. Sampling rate changes such as decimation (Section 5.6) act on the input in the same way, through the autocorrelation at each lag, and we expect the same approach to apply; however, we have not tested it.

### 5.8. Robustness, Distribution Shift, and Limitations

Four forms of distribution shift were tested, all in simulation. First, we tested imperfection magnitudes five times larger than in training (harsh preset, Table 3). ReconUNet degrades gracefully (Moderate: $0.61^{\circ }\to 2.14^{\circ }$; Crowded: $2.45^{\circ }\to 4.15^{\circ }$), whereas SubViT collapses ($8.72^{\circ }\to 14.85^{\circ }$ in Moderate). We attribute this to the large direct regressor overfitting the imperfection statistics of the corpus. Second, we tested SNR outside the [−10,10] dB training range (Figure 8). Above this range, the floors of the learned methods are flat, while below it all methods enter the threshold region together. The third and fourth tests covered fewer snapshots or decimated sampling (Section 5.6) and a different source bandwidth (Section 5.7), respectively. Both tests change the temporal statistics of the input, and both cause failures rather than graceful degradation unless the training distribution provides coverage. For the bandwidth, ReconUNet-BW shows that covering the tested range is sufficient within that range. By contrast, the Saturated scenario lies inside the training distribution and is a capacity limit rather than a shift. These tests characterize the behavior of the method under the parametric models of Section 2; they do not establish robustness to conditions outside those models.

Five limitations bound the results:

- *The network is a regularizer, not a calibrator.* It learns the expected clean covariance under its training prior (Section 3.5), but does not estimate the imperfections of a particular array or learn any realization-specific correction (Section 5.5). On clean single-source scenes, the residual floor set by the prior makes raw Root-MUSIC marginally more precise ($0.25^{\circ }$ against $0.29^{\circ }$ at 0 dB, mild preset). When calibration data are available, calibrating the manifold remains the correct first step, leaving the frontend with less mismatch to absorb.
- *Temporal statistics.* The multi-lag representation depends intrinsically on the temporal correlation of the sources, and the trained network depends on the source statistics and sampling rate seen in training. When trained at one bandwidth, it fails for wideband or white sources; when trained on a range of bandwidths, it does not fail within that range (Section 5.7). Decimated snapshots and fewer than roughly a hundred contiguous snapshots were not covered by training; under these conditions, it is worse than the raw estimator (Section 5.6).
- *Separation.* Because the proposed network was trained with a $10^{\circ }$ minimum separation, it resolves sources closer than about $8^{\circ }$ less reliably than the raw estimator.
- *Degrees of freedom.* Seven coherent arrivals on eight elements defeated every tested method.
- *Simulation.* All results in this paper are from simulation. Training and test imperfections come from the same parametric family (Toeplitz coupling, uniform gain/phase errors, and Gaussian position jitter). Non-Toeplitz coupling and other distributions were not tested. Transfer to measured arrays has not been demonstrated. Every performance statement in this paper is a statement about this particular simulation setting; whether the gains carry over to real arrays is yet to be shown. Endfire directions also remain intrinsically ill-conditioned for ULAs.

### 5.9. Computational Cost

Table 7 lists the size, training cost, and inference cost of every learned method as measured on one NVIDIA RTX 2000 Ada GPU (16 GB). ReconUNet has 323,070 parameters and 7.1 MMAC per scene, and trained in 8.9 h. SubspaceNet is smaller (41,761 parameters, 3.3 MMAC), but because it backpropagates through Root-MUSIC, it trained in 30.3 h. SubViT has 42.6M parameters and 382 MMAC (43.9 h). Each DA-MUSIC instance has 15,187 parameters and 0.8 MMAC, with four instances needed for K≤4 (2.6–7.7 h each, including the continuation). At inference, the frontend adds one convolutional pass per scene: 0.76 ms on the GPU at batch size 1, 0.014 ms per scene at batch size 1024, and 0.49 ms on a 20-thread CPU at batch size 1. Without the eigendecomposition heads, the network is about 35% faster than ReconUNet-EVD on the GPU and five times faster on the CPU. For comparison, Root-MUSIC on the raw covariance costs 0.26 ms per scene on the CPU, and the eigendecomposition of the 8×8 sample covariance alone costs 11 μs. Thus, at batch size 1, the frontend costs two to three times as much as the estimator it feeds, and costs less when batched. The backbone scales as $O(\tau N^{2})$ per layer, so the overhead remains modest for arrays of moderate size. As ReconUNet-BW has the same architecture, it has the same size and cost.

## 6. Conclusions

This paper has introduced ReconUNet, a learned frontend that reconstructs a clean covariance from multi-lag autocorrelations and passes it to unmodified classical DoA estimators. The network is a U-Net with a Hermitian covariance head, trained with a single reconstruction loss toward the closed-form clean covariance on the nominal manifold, with no estimator in the training loop. A controlled simulation study on identical data compares it with SubspaceNet, DA-MUSIC, a covariance input transformer regressor, six classical estimators, and spatial smoothing. A single trained model attained the lowest pooled error on the held-out split ($4.23^{\circ }$, against $7.91^{\circ }$ for the best learned baseline and $12.43^{\circ }$ for raw Root-MUSIC), the lowest error for every number of sources, and a median error equal to the best of any other method. The same weights served MUSIC, Root-MUSIC, ESPRIT, and Unitary-ESPRIT, and the gains held under simulated imperfections five times larger than those in training.

Two findings shape how the method should be understood. First, the simpler design is better; the eigendecomposition heads and the auxiliary subspace and eigenvalue losses of our first design did not help, while removing them improved every statistic, reduced the precision floor on easy scenes to within $0.04^{\circ }$ of the raw estimator, and made the network faster. Second, the network is a learned regularizer of the covariance under a prior, not an array calibrator; it learns no realization-specific correction of the imperfections and inherits the statistics of its training distribution, most importantly the temporal correlation of the sources, which gives the non-zero lags their information. Within that distribution, it removes the catastrophic failures that coherent multipath causes in subspace methods, a regime in which spatial smoothing fails on an array this small. Outside this distribution (for example for wideband sources when trained at one bandwidth, for decimated sampling, or for fewer than about a hundred snapshots), it can be worse than the raw estimator. Widening the training distribution mitigates this impact within the range covered by training. When trained on a range of source bandwidths, the same network was the most accurate method across that range and on a test set with randomly drawn bandwidths, at a small cost at the nominal bandwidth. However, the dependence of the multi-lag representation on the temporal correlation of the sources is intrinsic, and training can only cover it, not remove it.

The limitations are listed in Section 5.8, and include dependence on the temporal statistics seen in training, no resolution advantage below the training separation, a degrees-of-freedom limit at seven coherent arrivals on eight elements, and validation restricted to parametric imperfection models in simulation. The results should be read as a characterization of the method under the simulation settings; they do not demonstrate performance on measured data. Future work will validate the approach on measured array data, which is the decisive test of transfer from synthetic training to real arrays. In addition, future work is needed to widen the training distribution in separation, snapshot count, and sampling rate, as was done here for the bandwidth; to extend training to other array geometries; and to address source enumeration, which both the proposed method and the learned baselines assume to be known.

## Figures and Tables

**Table 1:** Positioning of ReconUNet relative to learned covariance/subspace front-ends. “Estimator in loop” means an estimator is differentiated through during training.

	SubspaceNet [25]	DA-MUSIC [26]	SDOA-Net [24]	Cov. Recon. Hybrid [28]	ReconUNet
Input	multi-lag autocorr.	sample covariance	snapshots	sample covariance	multi-lag autocorr.
Output	covariance surrogate	pseudo-cov. + spectrum	spectrum	covariance + angles	covariance
Supervision	angles (via Root-MUSIC)	angles (via MUSIC)	spectrum labels	angles	clean covariance
Estimator in loop	Root-MUSIC	MUSIC	none	angle head	none
Coherent sources	yes	yes	not explicit	not explicit	yes (rank-1 target)
Array imperfections	yes	not explicit	yes	not explicit	yes (randomized prior)
*K* required	yes	yes (fixed head)	no	fixed	yes (by the estimator)
Back-ends served	Root-MUSIC	MUSIC	peak search	own head	any spectral/subspace
Parameters	42 k	15 k per *K*	—	—	323 k

**Table 2:** Classical estimators on a perfect array (Basic scenario): RMSE in degrees at −10, 5, and 20 dB. The −10 dB column is in the threshold region.

Method	−10 dB	5 dB	20 dB
Bartlett	27.36	**0.207**	0.038
MVDR	26.81	0.208	0.092
MUSIC ($1^{\circ }$ grid)	33.70	0.236	0.197
Root-MUSIC	37.24	**0.207**	**0.032**
(Unitary-)ESPRIT	35.75	0.276	0.041

Bold values indicate the lowest RMSE in the 5 and 20 dB columns (equal values at the displayed precision are both marked); the −10 dB column is in the threshold region and is not marked.

**Table 3:** Pooled RMSE in degrees at 0 dB SNR, 1000 scenes per cell, for two imperfection presets; all learned models were trained on the mild preset. Blocks: classical estimators on the raw sample covariance; forward–backward spatial smoothing; classical estimators on the ReconUNet covariance (one trained model for all four back-ends); other learned frontends, including the ReconUNet-EVD variant. Best value per column in bold. Scenarios: Basic (1 source), Moderate (2+1 replica), Crowded (4+3), Saturated (1+6). Percentile-bootstrap 95% intervals (1000 resamples) are given in the text for the central comparisons and in the repository for every cell. The grid-MUSIC entries in the Basic column (raw and aided) are inflated by a few scenes in which the refined peak selection returns a spurious maximum (Section 5.1); their medians are $0.19^{\circ }$ and $0.24^{\circ }$.

Method	Mild Imperfections				Harsh Imperfections (5×)			
	Basic	Moderate	Crowded	Saturated	Basic	Moderate	Crowded	Saturated
Bartlett	3.96	12.92	21.00	28.72	6.14	14.50	21.83	30.22
MVDR	3.56	11.53	15.72	25.06	5.91	12.27	18.25	27.97
MUSIC	3.97	10.39	12.56	29.93	5.73	11.70	15.56	30.67
Root-MUSIC	**0.25**	5.91	8.87	40.95	**1.15**	6.83	9.34	41.96
ESPRIT	0.33	5.94	8.14	35.72	1.58	6.20	8.39	38.63
Unitary-ESPRIT	0.41	5.69	8.04	24.30	3.65	6.76	9.07	25.55
FBSS (L=5) + Root-MUSIC	—	8.54	12.42	—	—	—	—	—
FBSS (L=6) + Root-MUSIC	—	6.45	11.30	—	—	—	—	—
FBSS (L=7) + Root-MUSIC	—	6.31	9.61	—	—	—	—	—
ReconUNet + MUSIC	4.97	2.08	3.87	20.80	1.50	5.60	5.16	22.56
ReconUNet + Root-MUSIC	0.29	**0.61**	**2.45**	20.81	1.18	**2.14**	4.15	22.39
ReconUNet + ESPRIT	0.29	**0.61**	2.66	21.03	1.18	**2.14**	4.03	22.42
ReconUNet + Unitary-ESPRIT	0.29	**0.61**	2.49	**20.55**	1.18	**2.14**	**3.82**	**22.10**
ReconUNet-EVD + Root-MUSIC	0.37	1.79	4.80	22.89	1.20	2.36	6.14	25.31
SubspaceNet	0.54	2.92	7.91	21.49	1.28	4.73	8.82	23.24
SubViT	0.38	8.72	16.32	23.60	1.22	14.85	18.42	26.10
DA-MUSIC	2.13	3.60	6.65	24.28	2.74	4.59	7.50	25.52
CRLB (single source)	0.12	0.14	0.21	0.12	0.12	0.14	0.21	0.12

**Table 4:** Held-out test split (12,000 scenes; 3000 per *K*; SNR uniform in [−10,10] dB; mild imperfections; 81% of scenes with coherent replicas). Each cell: pooled RMSE with its percentile-bootstrap 95% interval, then the median per-scene RMSE, all in degrees. Both ReconUNet models use the Root-MUSIC backend; ReconUNet-EVD is the variant with eigendecomposition heads (Appendix A). Best pooled value per row in bold.

*K*	R-MUSIC (Raw)	ESPRIT (Raw)	SubspaceNet	SubViT	DA-MUSIC	ReconUNet-EVD	ReconUNet
1	26.37 [24.6, 28.0]; 1.14	23.45 [21.8, 25.2]; 4.94	13.62 [12.2, 15.1]; 1.42	13.87 [12.4, 15.4]; 0.53	16.25 [15.0, 17.6]; 4.09	12.49 [11.0, 14.0]; 0.75	**10.49** [8.9, 12.0]; 0.49
2	14.21 [13.3, 15.0]; 0.79	12.49 [11.4, 13.4]; 2.60	8.17 [7.4, 8.9]; 1.44	15.24 [14.3, 16.2]; 0.53	8.31 [7.7, 8.9]; 2.90	6.04 [5.1, 7.0]; 0.84	**4.17** [3.2, 5.1]; 0.52
3	9.71 [9.1, 10.4]; 0.77	8.79 [8.1, 9.5]; 1.82	7.57 [7.0, 8.0]; 1.74	15.92 [15.2, 16.6]; 0.59	6.38 [6.0, 6.8]; 3.15	3.73 [3.2, 4.3]; 0.98	**2.35** [1.8, 2.9]; 0.60
4	6.41 [5.9, 6.9]; 0.67	5.41 [4.9, 5.9]; 1.15	7.24 [6.9, 7.6]; 2.06	14.31 [13.8, 14.8]; 0.67	5.04 [4.8, 5.3]; 3.05	4.00 [3.5, 4.5]; 1.12	**2.11** [1.7, 2.5]; 0.66
all	12.43 [12.0, 12.9]; 0.80	11.00 [10.5, 11.5]; 2.15	8.37 [8.0, 8.7]; 1.70	14.95 [14.6, 15.3]; 0.58	7.91 [7.6, 8.2]; 3.17	5.79 [5.3, 6.2]; 0.94	**4.23** [3.8, 4.7]; 0.58

**Table 5:** Ablation under a reduced identical training protocol (10% of the corpus, 40 epochs; Root-MUSIC backend). Test split: pooled and median RMSE (degrees), relative covariance error, leading-*K* subspace distance, relative eigengap error (all from the eigendecomposition of $\hat{R}$). Right: pooled RMSE at 0 dB in the Moderate and Crowded scenarios. Best value per column in bold. The last row is trained on a single fixed imperfection realization; its error on the test split rendered with that same realization is $9.24^{\circ }$, against $9.25^{\circ }$ for the reference model on the same scenes.

Variant	Test Split (12,000 Scenes)					0 dB Scenarios	
	Pooled	Median	Cov. Err.	Subsp. Dist.	Gap Err.	Moderate	Crowded
ReconUNet-EVD (reference)	9.22	1.28	0.357	0.534	0.128	4.05	9.39
$L_{\mathrm{rec}}$ only	7.27	1.00	0.288	0.380	0.134	3.69	7.02
$L_{\mathrm{rec}}+L_{\mathrm{proj}}$	7.03	1.01	0.286	0.376	0.134	3.63	6.71
Full without $L_{\mathrm{dom}}$	9.43	1.20	0.365	0.504	0.121	4.15	10.00
Full without $L_{\mathrm{eig}}$	7.37	1.06	0.291	0.417	0.156	**1.85**	7.20
Single lag (τ=1)	9.87	1.27	0.361	0.551	0.140	6.08	9.54
ReLU instead of anti-rectifier	8.54	1.18	0.332	0.494	**0.105**	3.35	8.45
No EVD heads, $L_{\mathrm{rec}}$ only (=ReconUNet)	**6.05**	**0.85**	**0.217**	**0.265**	0.204	1.89	**4.71**
Imperfections fixed across scenes	9.27	1.27	0.355	0.531	0.119	4.54	9.74

**Table 6:** Dependence on the source bandwidth *B* (fraction of the sampling rate): pooled RMSE in degrees at 0 dB, mild imperfections, 1000 scenes per cell, and identical scene geometry and replica delays at every bandwidth. |r(7)| is the measured normalized source autocorrelation at the last lag of the input stack. ^†^ Training bandwidth for ReconUNet and all baselines; ReconUNet-BW is trained on bandwidths drawn between 0.01 and 0.40 and on white sources. Best value per scenario and row in bold.

$B/f_{s}$	|r(7)|	Moderate (2+1)					Crowded (4+3)				
		R-MUSIC	ReconUNet	ReconUNet-BW	SubspaceNet	DA-MUSIC	R-MUSIC	ReconUNet	ReconUNet-BW	SubspaceNet	DA-MUSIC
0.01	0.99	5.91	2.05	**0.96**	5.33	6.72	9.51	5.93	**5.57**	11.27	8.33
0.02	0.97	5.93	0.82	**0.81**	4.36	4.78	9.13	3.49	**3.39**	9.20	7.41
0.05 ^†^	0.83	5.91	**0.61**	0.78	2.92	3.60	8.87	**2.45**	2.57	7.91	6.65
0.10	0.39	5.67	1.65	**0.67**	3.73	3.33	8.80	3.01	**2.24**	8.58	6.14
0.20	0.23	5.91	22.70	**0.53**	5.87	3.12	8.78	16.67	**1.75**	10.74	5.83
0.40	0.09	5.63	26.42	**0.53**	8.10	2.98	8.02	19.88	**2.27**	11.61	5.41
white	0.04	5.27	30.88	**0.53**	11.64	2.84	7.56	20.52	**2.81**	17.56	5.55

**Table 7:** Model size and cost. Val. RMSPE: validation angular error of the selected checkpoint. Latency: median per scene, GPU (RTX 2000 Ada) unless stated, lag stack formation included where the method needs it; the two ReconUNet models were re-measured in the same session. MMAC: multiply-accumulates per scene in convolutional, linear, and recurrent layers (eigendecompositions and spectrum scans excluded). Training time on one GPU; DA-MUSIC K≥2 includes the continuation to 600 epochs.

Model	Params	Epochs (Best)	Val. RMSPE	Train Time	Latency, Batch 1	Latency, Batch 1024	MMAC
ReconUNet (network only)	323,070	207 (182)	$1.33^{\circ }$	8.9 h	0.76 ms	0.014 ms	7.1
ReconUNet + Root-MUSIC					1.33 ms	0.058 ms	
ReconUNet (network only, CPU, 20 thr.)					0.49 ms	—	
ReconUNet-EVD (network only)	337,842	109 (84)	$2.17^{\circ }$	4.8 h	1.16 ms	0.032 ms	7.8
ReconUNet-EVD (network only, CPU, 20 thr.)					2.55 ms	—	
SubspaceNet + Root-MUSIC	41,761	83 (58)	$3.99^{\circ }$	30.3 h	1.84 ms	0.72 ms	3.3
SubViT (D=768)	42,642,674	161 (136)	$6.44^{\circ }$	43.9 h	1.53 ms	0.14 ms	382
DA-MUSIC, K=1	15,187	207 (182)	$8.21^{\circ }$	2.6 h	0.76 ms	0.013 ms	0.8
DA-MUSIC, K=2	15,187	491 (466)	$4.98^{\circ }$	6.3 h			
DA-MUSIC, K=3	15,187	600 (595)	$4.39^{\circ }$	7.7 h			
DA-MUSIC, K=4	15,187	582 (557)	$3.89^{\circ }$	7.5 h			
Root-MUSIC on the raw covariance (CPU)	—	—	—	—	0.26 ms	0.097 ms	—
EVD of an 8×8 matrix (CPU)	—	—	—	—	0.011 ms	0.003 ms	—

## Data Availability

The datasets used in this study are synthetic and are regenerated deterministically from the scene manifests and seeds included in the released code. The source code (data generation, training of ReconUNet and all learned baselines, classical estimators, and the evaluation harness that reproduces every table and figure), together with the trained model weights, is publicly available at https://github.com/itamaralmog96/ReconUNet-DoA (accessed on 1 November 2026).

## Appendix A. The ReconUNet-EVD Variant

The first version of this method, denoted ReconUNet-EVD here, added two heads to the network in Figure 4 and trained it with a four-term loss. It is reported in Section 5 as a reference, and its components are evaluated in the ablation of Section 5.5.

Heads

An eigenvalue head applies global average pooling over the 2N×N plane, followed by an MLP, producing non-negative eigenvalues $\hat{\lambda }\in R_{+}^{N}$ sorted in descending order. An eigenvector head (a shallow CNN) then predicts ${\hat{U}}_{\mathrm{raw}}\in C^{N\times N}$, which is projected onto the Stiefel manifold by a differentiable complex QR factorization $\left[\hat{U},\;\cdot \;\;\right]=\mathrm{cQR}\left({\hat{U}}_{\mathrm{raw}}\right)$ so that ${\hat{U}}^{H}\hat{U}=I_{N}$. Each column is defined only up to a unit-modulus phase, which is fixed by rotating the column so that its first entry is real and positive.

Loss

With $R^{★}=U^{★}\mathrm{diag}\left(\lambda^{★}\right){\left(U^{★}\right)}^{H}$ being the eigendecomposition of the target (17), the variant minimizes

(A1) $$L_{\mathrm{EVD}}=L_{\mathrm{eig}}+L_{\mathrm{proj}}+L_{\mathrm{dom}}+L_{\mathrm{rec}},$$

with unit weights, where $L_{\mathrm{rec}}$ is (18), $L_{\mathrm{eig}}=\frac{1}{N}{∥\hat{\lambda }-\lambda^{★}∥}_{2}^{2}$, and with $U_{K}^{★},{\hat{U}}_{K}$ the leading *K* columns and $u_{1}^{★},{\hat{u}}_{1}$ the leading column,

(A2) $$L_{\mathrm{proj}}=\frac{1}{N^{2}}∥{\hat{U}}_{K}{\hat{U}}_{K}^{H}-U_{K}^{★}{\left(U_{K}^{★}\right)}^{H}{∥}_{F}^{2},\;L_{\mathrm{dom}}=1-|{\hat{u}}_{1}^{H}u_{1}^{★}|.$$

Here, $L_{\mathrm{proj}}$ is the squared projection-Frobenius distance between the signal subspaces, which is invariant to per-column phase and rotations within the subspace, while $L_{\mathrm{dom}}$ is invariant to the phase ambiguity of a complex eigenvector. The first submitted version of this paper misstated $L_{\mathrm{dom}}$ as a sign-robust Euclidean distance; (A2) is the form that is actually implemented and used.

Why the first version was abandoned

For an 8×8 matrix, a numerical EVD costs about 11 μs (Table 7), so predicting the eigendecomposition gives no runtime advantage. The intended purpose was for the loss to act directly at training time on the quantities used by MUSIC and ESPRIT. However, as the ablation of Section 5.5 shows, this does not work as intended. With unit weights, $L_{\mathrm{eig}}$ dominates (A1) by an order of magnitude, the covariance is reconstructed less accurately, and the estimator receives a worse input. Using the predicted eigenbasis directly gives the same estimates as the numerical EVD of the reconstructed covariance.
